# Supplementary material for: Extent of N-terminus exposure of monomeric alpha-synuclein determines its aggregation propensity
Source: Nat Commun. 2020 Jun 4;11:2820. doi: 10.1038/s41467-020-16564-3 (PMC7272411; doi:10.1038/s41467-020-16564-3)
Supplement: Supplementary file 1 — Supplementary Information [file 41467_2020_16564_MOESM1_ESM.pdf]

Supplementary Information
Extent of N-terminus exposure of monomeric alpha-synuclein determines its aggregation propensity

Amberley D. Stephens, Maria Zacharopoulou, et al.,

Contents

Supplementary Figure 1. ThT-based aggregation assay reveals that D121A aSyn does not aggregate to the same extent as WT, D115A and D119A upon incubation with calcium. 5

Supplementary Figure 2. AFM reveals D121A aSyn forms mostly oligomeric structures, while WT, D115A and D119A aSyn form fibrillary structures upon incubation with CaCl2................................. 6

Supplementary Note 1: Discussion of aSyn D to A mutant ThT assays 7

Supplementary Figure 3. Full zoomed out spectrum of ^{1}H-^{15}N aSyn WT compared to aSyn D121A and pS129. 8

Supplementary Figure 4. aSyn pS129 and D121A have different chemical shifts in increasing calcium concentrations from 0.2 – 4.2 mM... 9

Supplementary Figure 5. ^{1}H-^{15}N spectra of aSyn WT in the presence or absence of 4 mM NaCl shows no difference in chemical shifts. 10

Supplementary Figure 6. Labelled ^{1}H-^{15}N spectra in the presence or absence of calcium of D121A aSyn. 11

Supplementary Figure 7. Labelled ^{1}H-^{15}N spectra in the presence or absence of calcium of pS129 aSyn. 12

Supplementary Figure 8. Dissociation constants of WT, pS129 and D121A aSyn and calcium reveal no significant differences between the different variants compared to WT aSyn and positive cooperativity of calcium binding to aSyn.. 13

Supplementary Figure 9. ThT-based aggregation assay reveals that D121A aSyn and pS129 aSyn do not aggregate as fast as WT aSyn... 14

Supplementary Figure 10. Representative chromatograph of the remaining aSyn monomer concentration as determined at the end of the ThT-based assay and analysed by size exclusion chromatography.. 15

Supplementary Table 1. p-Values from unpaired t-test for the comparison of protein states WT, D121A. 16

Supplementary Table 2. p-Values from unpaired t-test for the comparison of protein states WT, pS129. 17

Supplementary Table 3. p-Values from unpaired t-test for the comparison of protein states WT, WT + Ca2+ 18

Supplementary Table 4. p-Values from unpaired t-test for the comparison of protein states D121A, D121A + Ca2+... 19

Supplementary Table 5. p-Values from unpaired t-test for the comparison of protein states pS129, pS129 + Ca2+... 20

Supplementary Figure 11. Peptide coverage map of aSyn using HDX-MS. 21

Supplementary Figure 12. ThT-based aggregation assay reveals that the aSyn familial mutants display different aggregation behaviour upon the addition of calcium. 22

Supplementary Figure 13. Native nano ESI-MS spectra of WT aSyn with increasing concentrations of calcium.. 23

Contents continued

Supplementary Figure 14. Native nano ESI-MS spectra of unbound aSyn and 1:10 and 1:20 aSyn to calcium ratios for aSyn mutants. 24

Supplementary Figure 15. Analysis of number of Ca^{2+} ions bound to the D to A aSyn mutants 1:10 and 1:50 protein to calcium ratios reveals no significant differences between the different aSyn variants. 25

Supplementary Figure 16. Nano-ESI-IM-MS reveals several aSyn conformations in equilibria and compaction upon addition of calcium. 26

Supplementary Table 6. Average percentage of the distribution of conformations of WT aSyn and mutants determined by nano-ESI-IM-MS in the absence calcium shows small changes in percentage distribution of conformations between the aSyn and its variants. 28

Supplementary Table 7. Average percentage of the distribution of conformations of WT and aSyn mutants determined by nano-ESI-IM-MS in the presence of calcium shows significant differences upon the addition of calcium to aSyn and its variants. 28

Supplementary Figure 18. CCS values of aSyn WT and familial mutants show multiple coexisting conformations and extensions at higher charge states....29

Supplementary Figure 19. CCS values of aSyn WT and familial mutants bound to two calcium ions show multiple coexisting conformations....30

Supplementary Note 2: Discussion of nano-ESI-IM-MS....31

Supplementary Table 8. p-Values from unpaired t-test for the comparison of protein states WT, A53T....32

Supplementary Table 9. p-Values from unpaired t-test for the comparison of protein states WT, A53E....33

Supplementary Table 10. p-Values from unpaired t-test for the comparison of protein states A53T, A53E....34

Supplementary Table 11. p-Values from unpaired t-test for the comparison of protein states A53T, A53T + Ca2+....35

Supplementary Table 12. p-Values from unpaired t-test for the comparison of protein states A53E, A53E + Ca2+....36

Supplementary Figure 20. Analysis of WT and $^{13}C/^{15}N$ pS129 aSyn purity and percentage phosphorylation. 37

Supplementary Figure 21. Deuterium uptake plots, measured in Dalton (Da), for representative peptides containing aar 4-17 (N-terminus) and aar 125-132 (C-terminus) of WT aSyn in the presence (red) and absence (blue) of calcium. 38

Supplementary Figure 22. Deuterium uptake plots, measured in Dalton (Da), for WT aSyn in the presence (red) and absence (blue) of calcium, showing the individual replicates for each measurement. 39

Supplementary Figure 23. Deuterium uptake plots, measured in Dalton (Da), for A. D121A aSyn in the presence (red) and absence (blue) of calcium, and B. pS129 aSyn in the presence (red) and absence (blue) of calcium, showing the individual replicates for each measurement....40

Supplementary Figure 24. Deuterium uptake plots, measured in Dalton (Da), for A. WT aSyn (blue) and D121A aSyn (red), and B. WT aSyn (blue) and pS129 aSyn (red), showing the individual replicates for each measurement. 41

Contents continued

Supplementary Figure 25. Deuterium uptake plots, measured in Dalton (Da), for A. A53T aSyn in the presence (red) and absence (blue) of calcium, and B. A53E aSyn in the presence (red) and absence (blue) of calcium, showing the individual replicates for each measurement. 42

Supplementary Figure 26. Deuterium uptake plots, measured in Dalton (Da), for A. WT aSyn (blue) and A53T aSyn (red), and WT aSyn (blue) and A53E aSyn (red), showing the individual replicates for each measurement. 43

Supplementary Figure 27. Deuterium uptake plots, measured in Dalton (Da), for A53T aSyn (blue) and A53E aSyn (red), showing the individual replicates for each measurement. 44

Supplementary Methods 45

Supplementary Table 13. Sequences of primers used in this study 46

Supplementary References 47

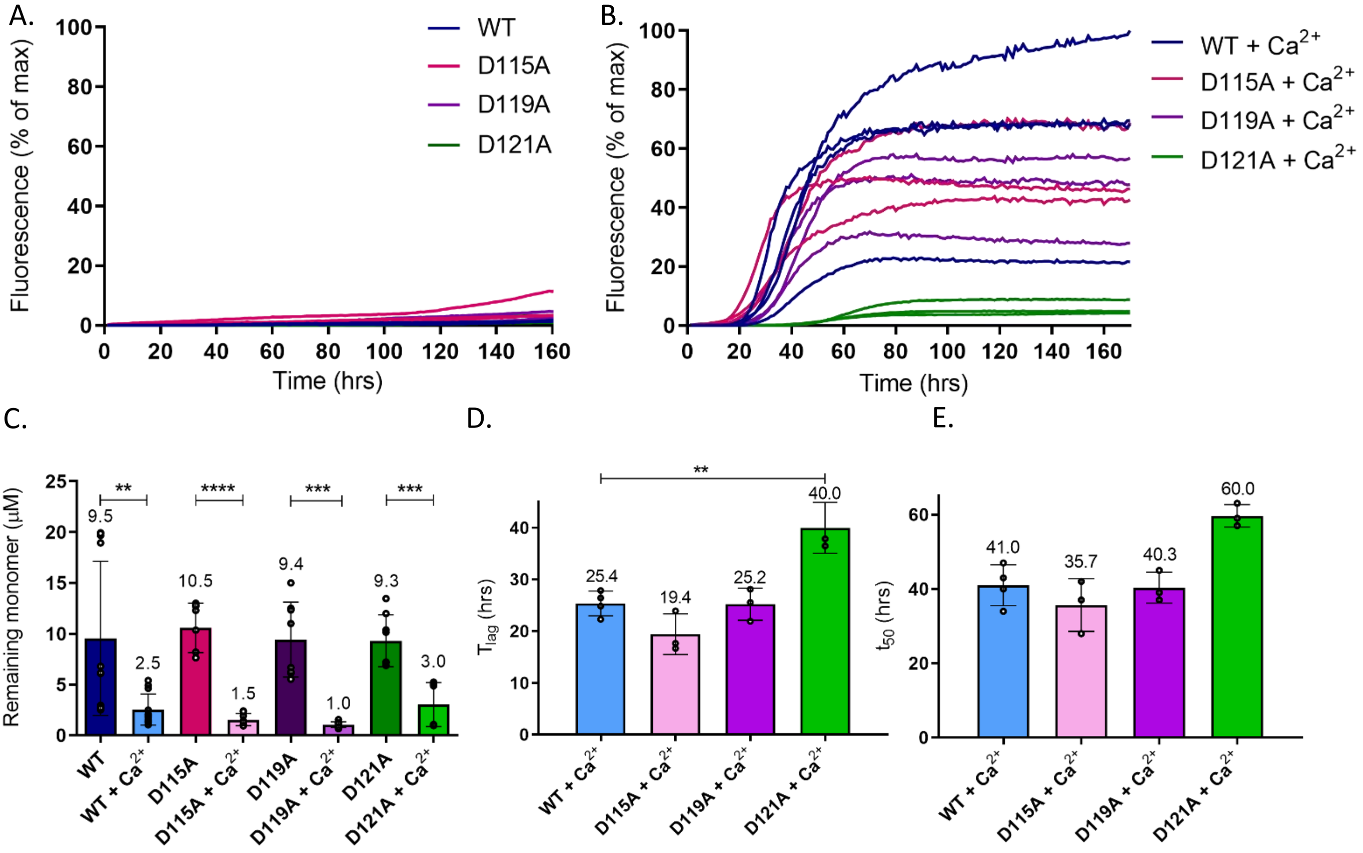

Supplementary Figure 1. ThT-based aggregation assay reveals that D121A aSyn does not aggregate to the same extent as WT, D115A and D119A upon incubation with calcium

(A.) The average ThT fluorescence intensity of independent experiments is plotted as % of maximum fluorescence per plate of WT (blue), D115A (green), D119A (purple) and D121A aSyn (red) and (B.) in the presence of 2.5 mM $CaCl_2$. 25 µM aSyn in Tris pH 7.2 was incubated with 10 µM ThT in a 384 well plate with orbital agitation at 300 rpm for 5 minutes before each read every hour for 160 hours. (C.) The emaining monomer concentration was measured using SEC-HPLC, 35 µL of monomer from each well in the ThT assay was analysed on an AdvanceBio SEC 130Å column equilibrated in 20 mM Tris pH 7.2 at 1 mL min^{-1}. Remaining monomer concentration was measured from the area under the peak and calculated using a standard curve of known concentrations. The mean remaining monomer concentration is numerically shown and for WT**p=0.0086, D115A****p<0.0001, D119A***p=0.0004, D121A***p=0.0001. For WT aSyn 12 wells per condition were measured, for D to A aSyn mutants eight wells per condition were measured. (D.) Lag time (T_{lag}) and (E.) time to reach 50 % of maximum aggregation (t_{50}) could only be calculated for assays containing calcium as there was no clear aggregation plateau in aSyn samples without calcium. The mean of three experiments is numerically shown and for (D.) **p<0.0026. Measurements were repeated with at least four sample replicates in three experiments, for each experiment the mean is represented by a circle in (D,E), and each individual well measured is represented by a circle in (C). An unpaired t test with Welch's correction assuming unequal s.d. was used to determine statistical differences. Error bars represent s.d.

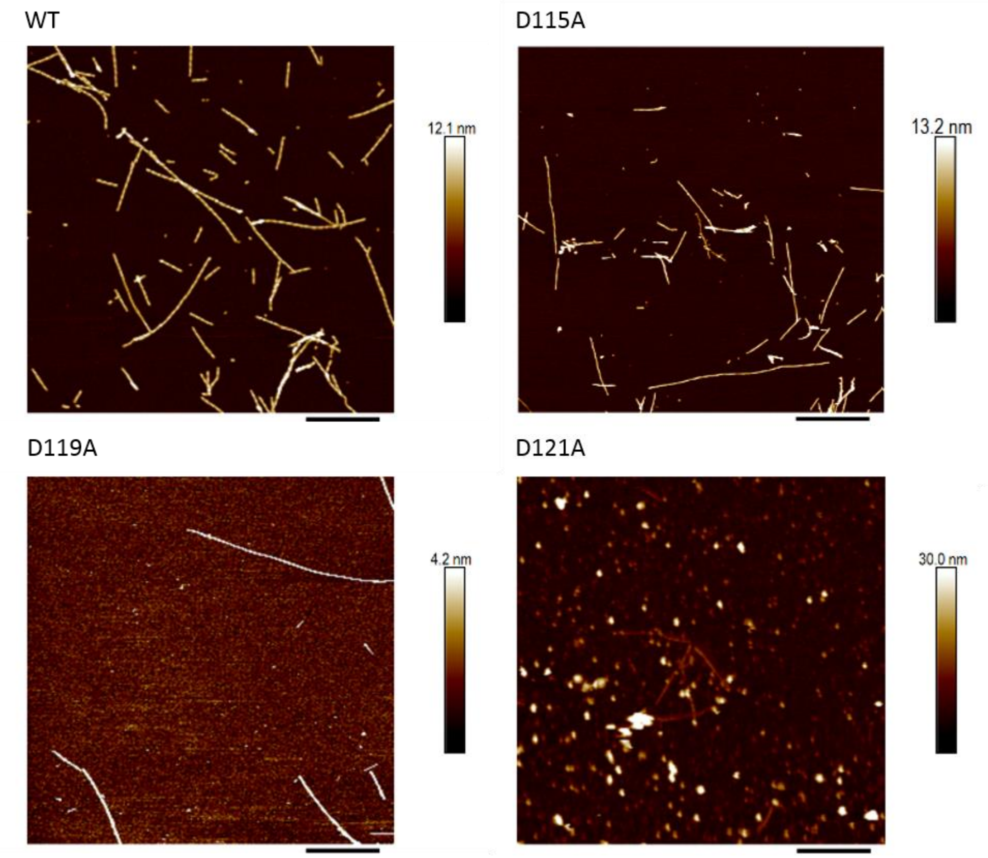

Supplementary Figure 2. AFM reveals D121A aSyn forms mostly oligomeric structures, while WT, D115A and D119A aSyn form fibrillary structures upon incubation with $CaCl_2$
aSyn samples were taken after ThT-based assays and incubated on freshly cleaved mica coated in 0.01% poly-lysine. Samples were washed in dH_2O and dried. Representative images show fibril formation for WT, D115A and D119A aSyn samples, but mostly oligomeric structures formed in D121A aSyn samples in the presence of 2 mM $CaCl_2$. At least four fields of view were imaged, more images are available at the Cambridge University Repository. Scale bar = 800 nm.

Supplementary Note 1

Discussion of aSyn D to A mutant ThT assays

Thioflavin T (ThT)-based kinetic assays showed little difference in aggregation rates for D115A, D119A, D121A and WT aSyn in the absence of calcium, but in the presence of calcium the D121A aSyn mutant had a significantly reduced aggregation rate compared to the other aSyn variants (Supplementary Figure 1). Furthermore, D121A aSyn did not aggregate into fibrillary structures under these conditions as determined by AFM (Supplementary Figure 2), suggesting that D121A may not bind calcium or has a different monomeric structure which appears to form oligomers, but does not readily elongate into fibrils.

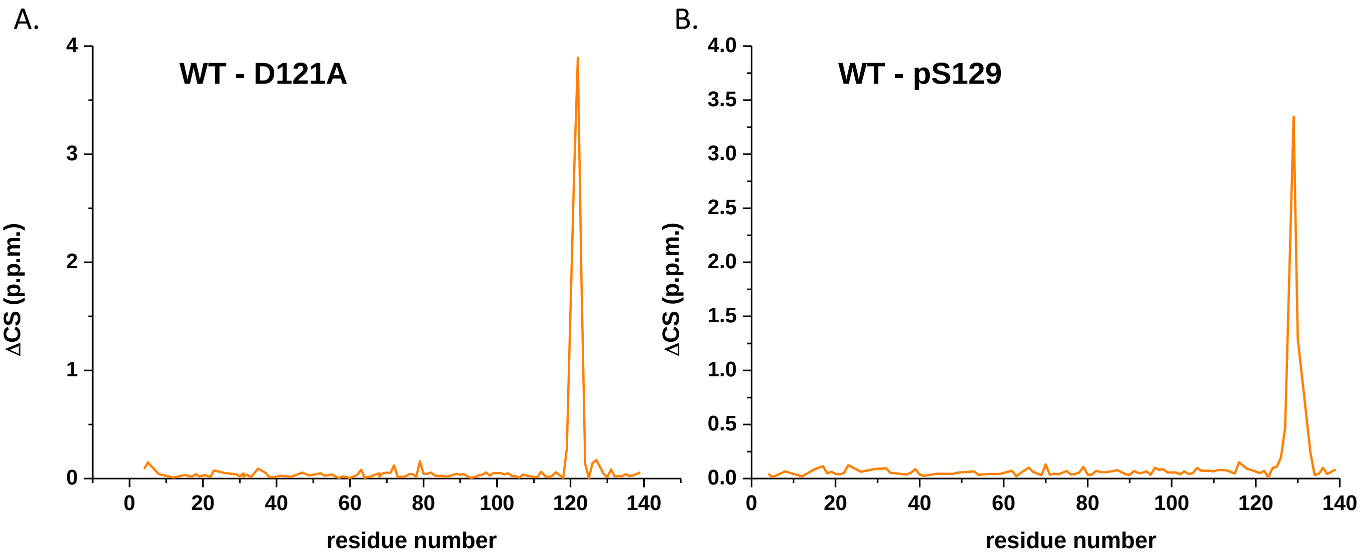

Supplementary Figure 3. Full zoomed out spectrum of ^{1}H-^{15}N aSyn WT compared to aSyn D121A and pS129

(A) We compared chemical shift perturbations in the amide backbone of ^{1}H-^{15}N D121A aSyn to WT aSyn (A) and pS129 aSyn to WT aSyn (B). The full spectrum shows clear CSPs around the location of the D to A mutation and the PO_4^- of S129 at the C-terminus, but information for the remaining residues is lost on this scale, see Figure 2 of main text.

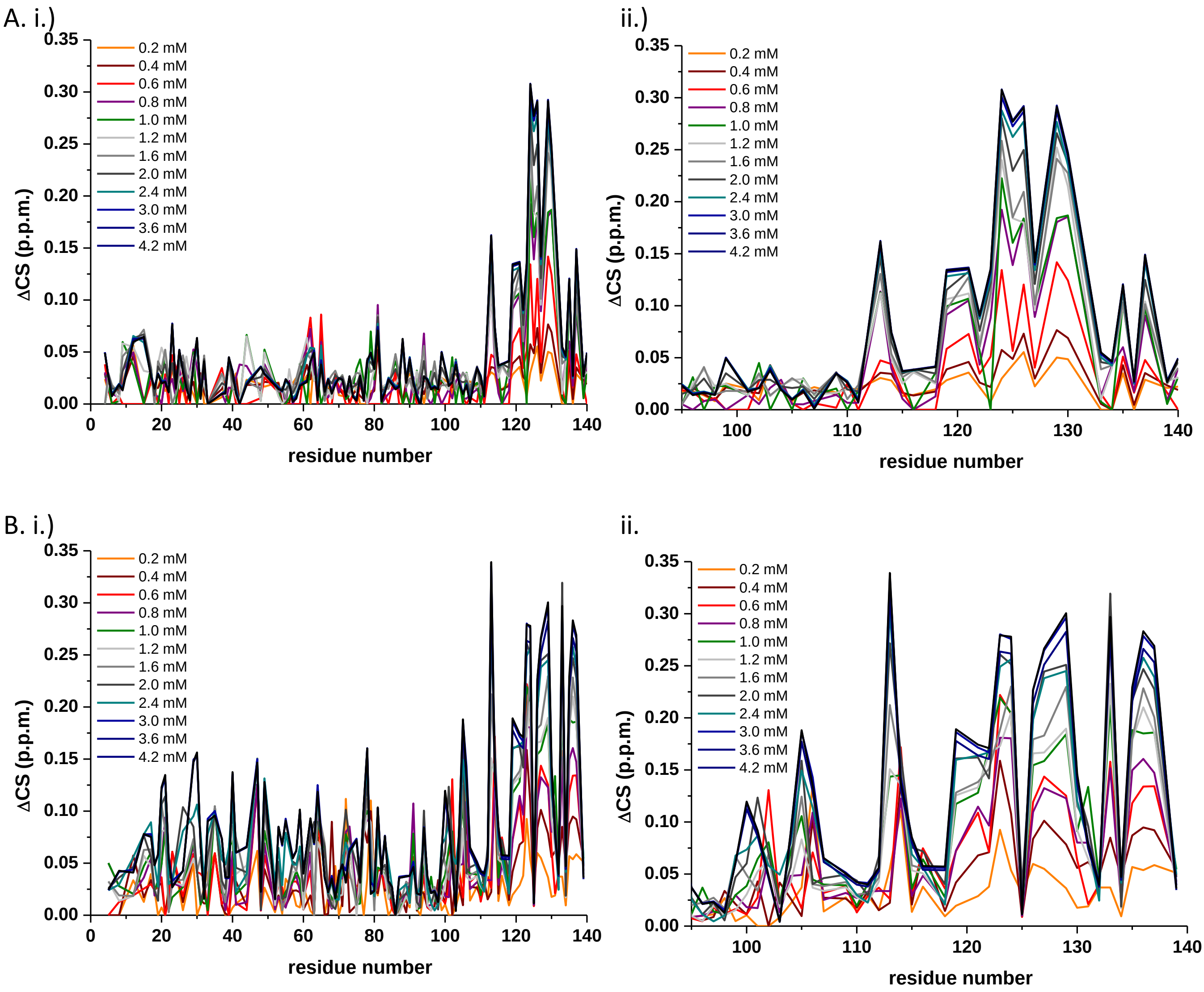

Supplementary Figure 4. aSyn pS129 and D121A display different chemical shift pertubations at increasing calcium concentrations ranging from 0.2 – 4.2 mM

$^{1}H^{15}N$ HSQC NMR spectra were measured at increasing calcium concentrations, from 0.2 mM to 4.2 mM. Chemical shift changes for the full length aSyn pS129 (A.i.) and zoomed in for amino acids 110 to 140 (A.ii.) and for the aSyn D121A full length protein (B.i.) and zoomed in for amino acids 110 to 140 (B.ii.) are plotted against the residue number. Saturation of signal changes occurred at ~3.6 mM calcium. Data for calcium titrations with WT aSyn can be found in the SI of Lautenschlaeger, et al., Nat. Comms. (2018) 9:712

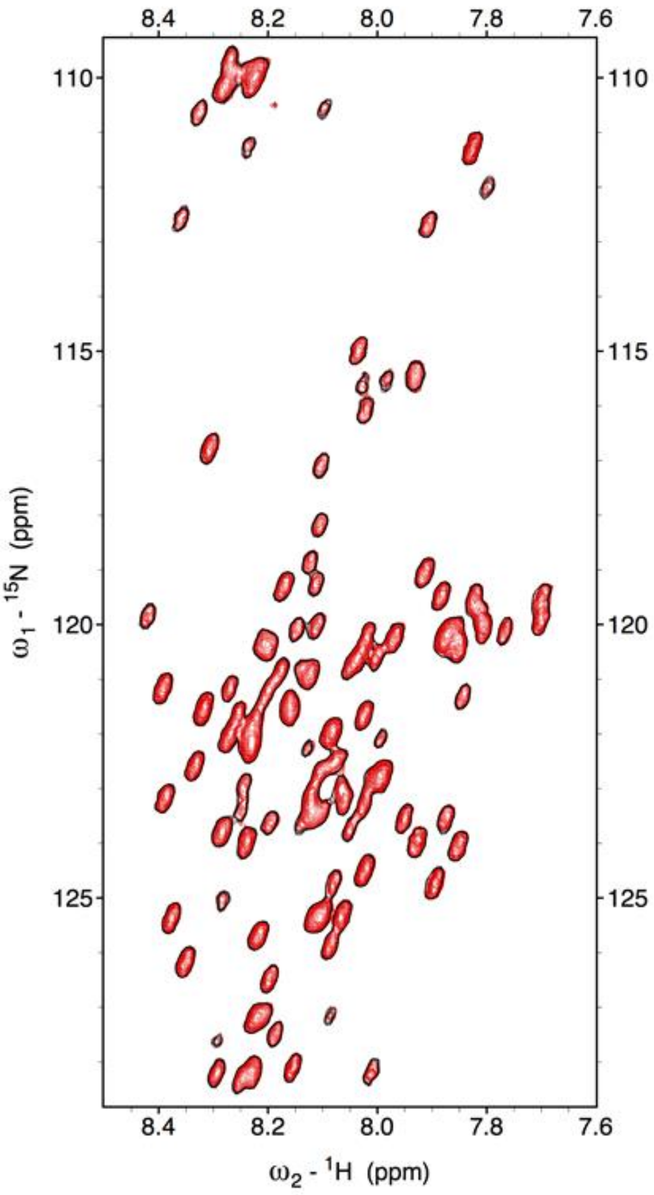

Supplementary Figure 5. ^{1}H-^{15}N spectra of aSyn WT in the presence or absence of 4 mM NaCl shows no difference in chemical shifts perturbations.
aSyn WT (200µM) in Tris Buffer 7.2 pH with no salt (red) and with 4 mM NaCl (black). The two spectra fully overlap, so for clarity the HSQC measured at 4 mM NaCl is shown as a single black contour line.

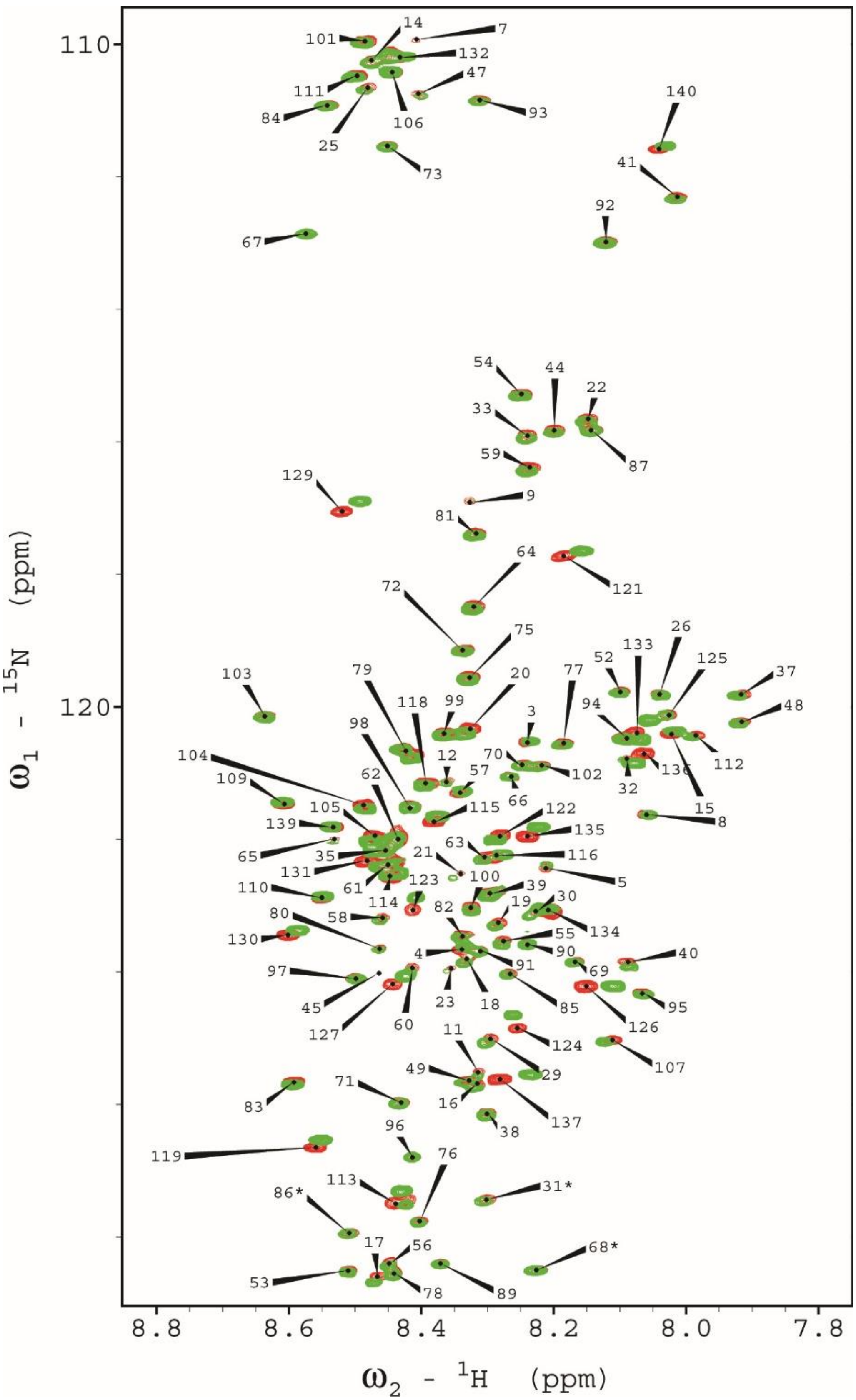

Supplementary Figure 6. Labelled ^{1}H-^{15}N spectra in the presence or absence of calcium of D121A aSyn 2D ^{1}H-^{15}N HSQC NMR spectra of D121A aSyn in the absence (red) and in the presence of calcium (green). Major chemical shift perturbations in the presence of calcium are located at the C terminus.

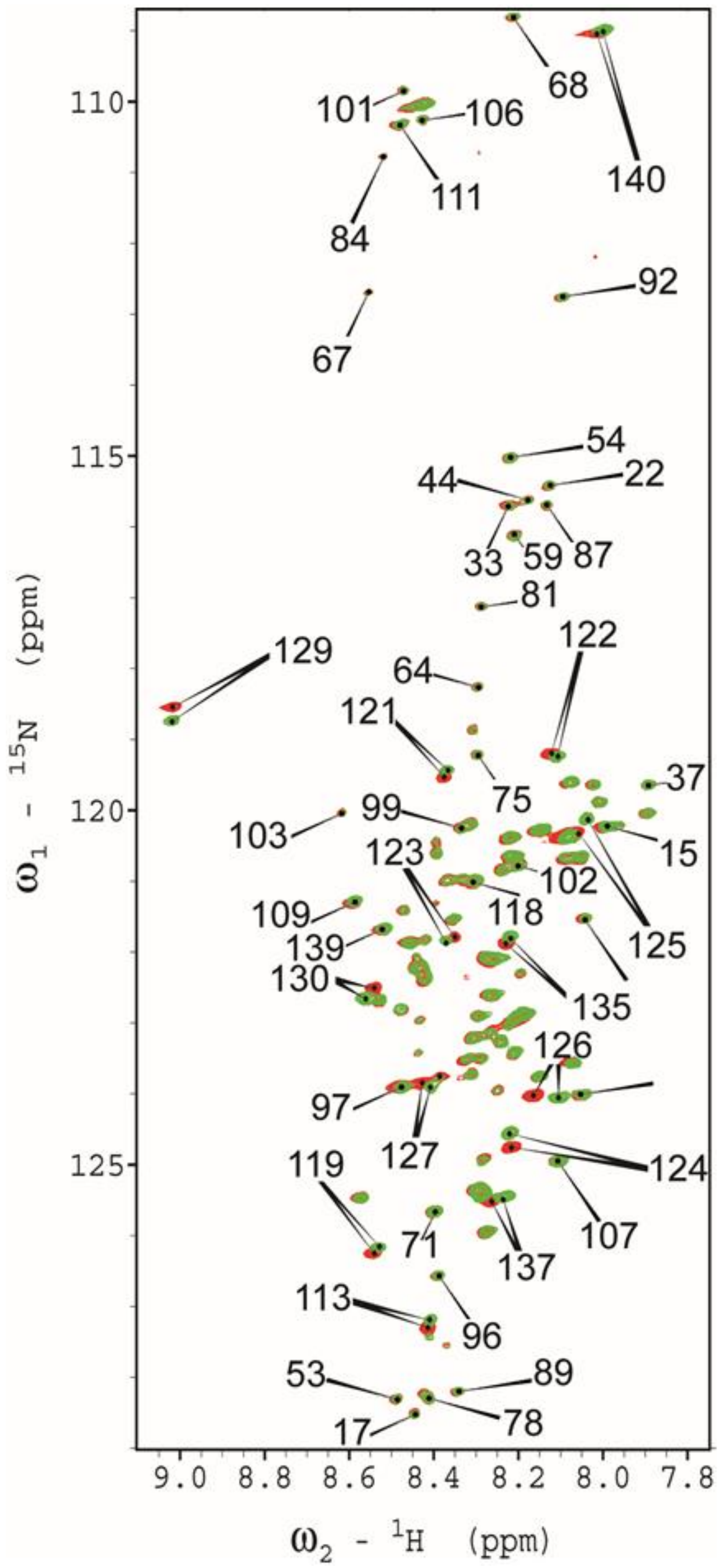

Supplementary Figure 7. Labelled ^{1}H-^{15}N spectra in the presence or absence of calcium of pS129 aSyn 2D ^{1}H-^{15}N HSQC NMR spectra of pS129 aSyn in the absence (red) and in the presence of calcium (green). Major chemical shift perturbations in the presence of calcium are located at the C terminus.

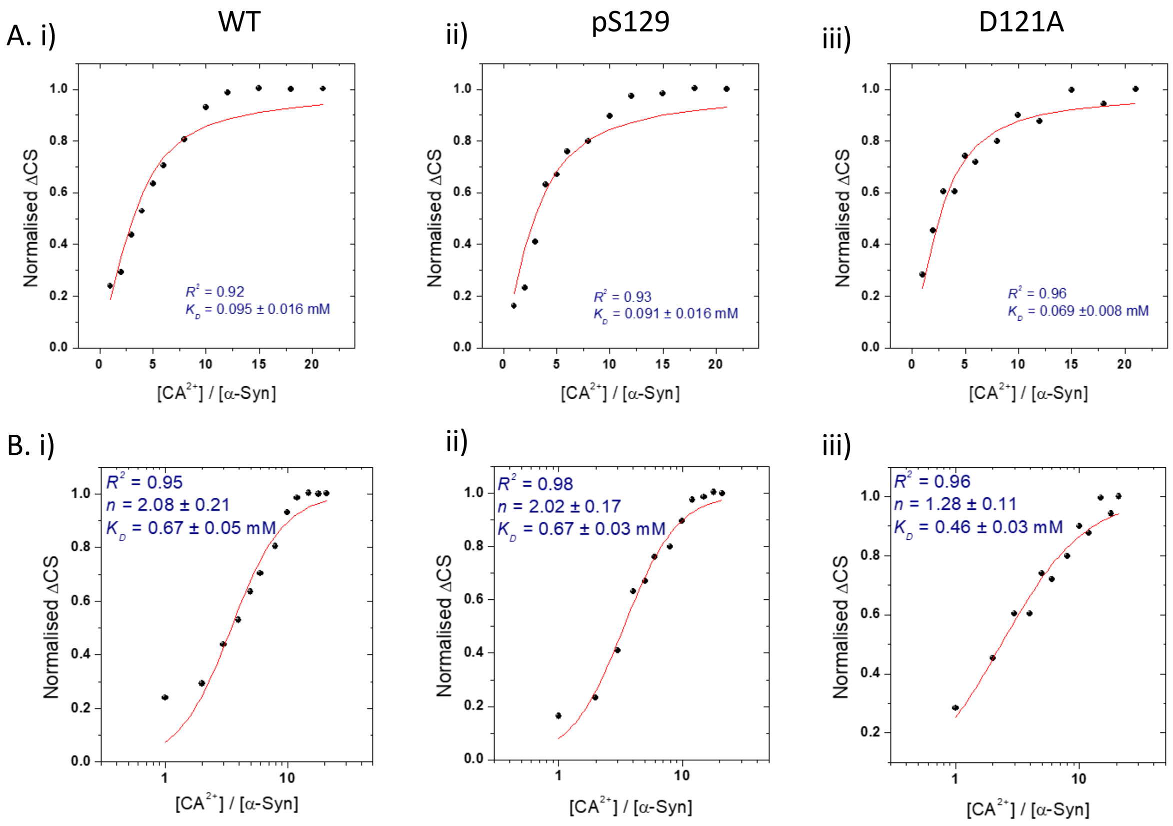

Supplementary Figure 8. Dissociation constants of WT, pS129 and D121A aSyn and calcium reveal no significant differences between the different variants compared to WT aSyn and display positive cooperativity of calcium binding to aSyn

^{1}H-^{15}N HSQC spectra of WT, pS129 and D121A aSyn (200 µM) was collected in the presence of increasing concentrations of Ca^{2+} (0.0 mM to 4.2 mM). (A) Fitting of aSyn calcium binding (K_D), where the number of calcium ions bound is fixed to 3, based on MS data. Calculated K_D were fitted for i) WT, ii) pS129, iii) D121A aSyn. (B) A second fitting was then used which takes into account cooperative binding using the Hill equation and revealed K_D values for i) WT, ii) pS129, iii) D121A aSyn. For all fittings using the Hill equation the n values are larger than 1 which shows positive cooperativity for the binding of calcium ions.

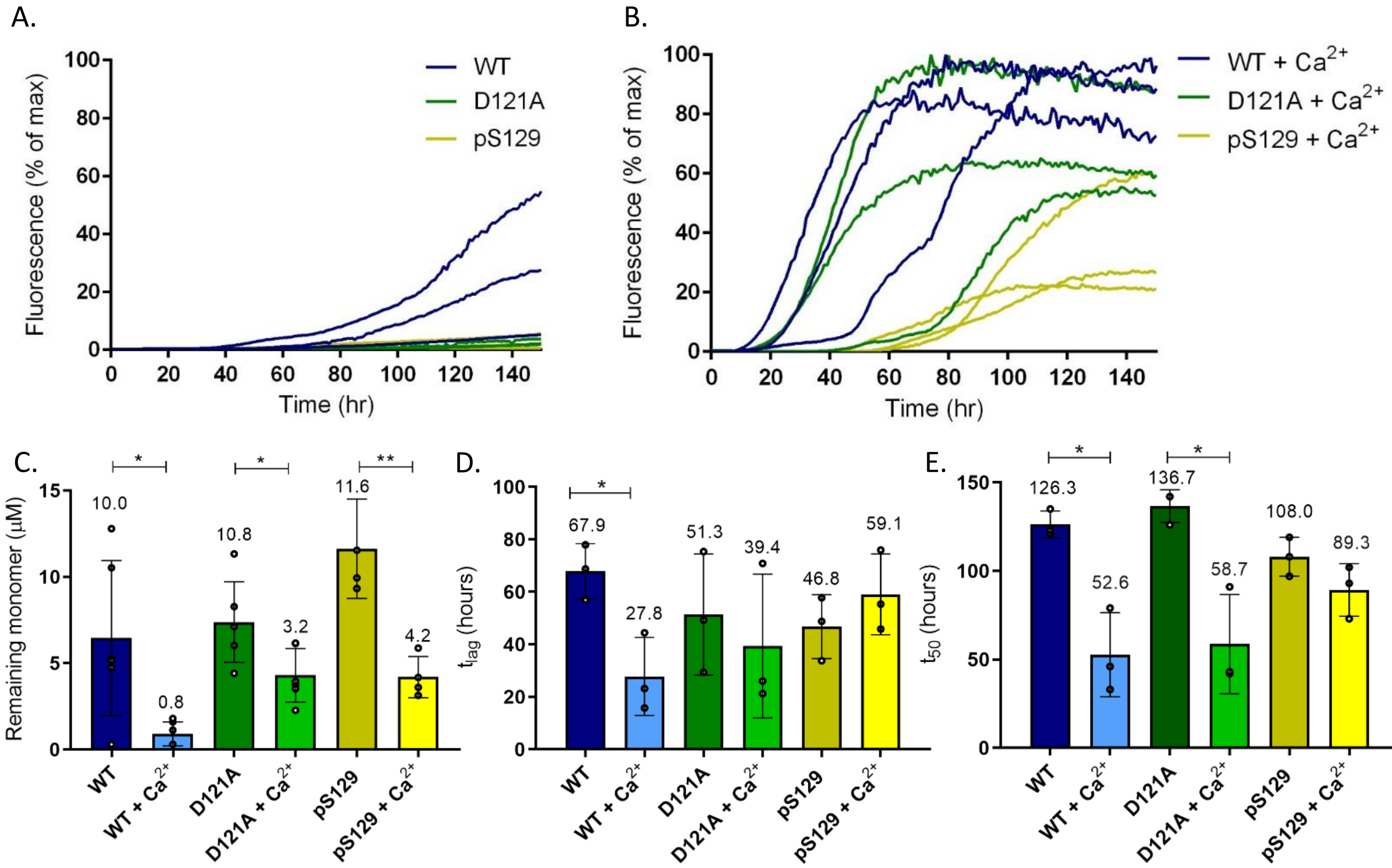

Supplementary Figure 9. ThT-based aggregation assay reveals that D121A aSyn and pS129 aSyn do not aggregate as fast as WT aSyn

(A.) The average ThT fluorescence intensity of independent experiments is plotted as % of maximum fluorescence per plate of WT (blue), D121A (green) and pS129 aSyn (yellow) and (B.) in the presence of 2.5 mM $CaCl_2$. 20 µM aSyn was incubated with 20 µM ThT in a half area 96 well plate with orbital agitation at 300 rpm for 5 minutes before each read every hour for 150 hours. (C.) The remaining monomer concentration was measured using SEC-HPLC, 35 µL of monomer from wells in the ThT assay were analysed on an AdvanceBio SEC 130Å column equilibrated in 20 mM Tris pH 7.2 at 1 mL min^{-1}. The remaining monomer concentration was measured from the area under the peak and calculated using a standard curve of known concentrations. The mean remaining monomer concentration is numerically shown and *p=0.0285 for WT, *p=0.0253 for D121A and **p=0.0088 for pS129 aSyn. For WT and D121A aSyn six wells per condition were measured, for pS129 four wells per condition were measured. (D.) Lag time (T_{lag}) and (E.) time to reach 50 % of maximum aggregation (t_{50}) were calculated and the mean numerically shown. For (D.) WT*p=0.023 and (E.) WT*p=0.0242 and D121A*p=0.0304. Measurements were repeated using at least four sample replicates from three experiments, each experiment mean is represented by a circle in (D,E), and each individual well measured is represented by a circle in (C.). An unpaired t test with Welch's correction assuming unequal s.d. was used to determine statistical differences. Error bars represent s.d.

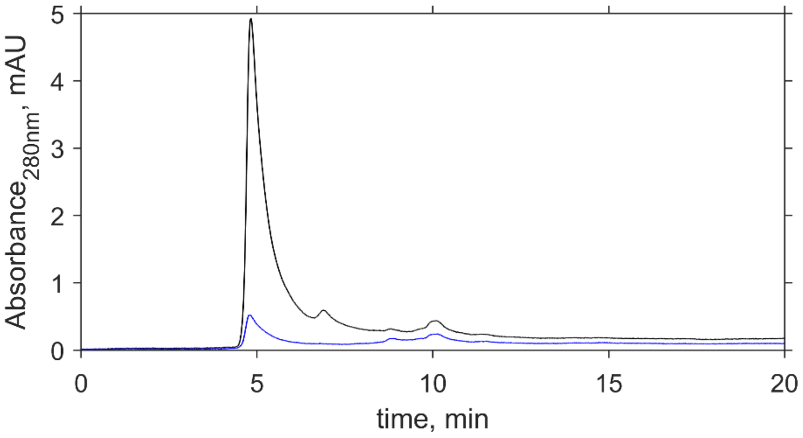

Supplementary Figure 10. Representative chromatograph of the remaining aSyn monomer concentration as determined at the end of the ThT-based assay and analysed by size exclusion chromatography

aSyn samples taken from the wells of ThT-based assays were centrifuged to remove fibrils and 35 µL of the remaining monomer was analysed by HPLC-SEC. 35 µL was injected on to an AdvanceBio SEC 130Å column at a flow rate of 1 ml/min in 20 mM Tris pH 7.2 and absorbance measured at 280 nm. The area under the curve reflects the remaining monomer concentration, which is calculated using known protein standards. The representative remaining monomer concentration from one well of WT aSyn (black) and WT aSyn + 2.5 mM $CaCl_2$ (blue) is shown.

Supplementary Table 1. p-Values from unpaired t-test for the comparison of protein states WT, D121A

WT vs D121A		
**Peptide**	**Discovery**	**p-Value**
1-17		6.35E-01
4-17		2.65E-02
5-17		2.85E-01
5-38		1.33E-01
18-38		1.41E-01
18-54		2.27E-01
39-54		1.94E-01
39-56		8.18E-01
39-57		3.94E-02
39-61		2.11E-02
39-69		3.88E-02
56-70		2.55E-02
55-89		7.13E-02
55-94		4.07E-01
70-89		1.25E-01
76-94		7.55E-01
77-94		7.36E-01
90-113		3.62E-01
94-113		4.31E-01
95-113		1.33E-01
124-134		5.04E-01
125-134		3.82E-01
124-140		5.09E-01
125-140		8.02E-01
132-140		1.23E-01
133-140		5.18E-01
135-140		1.71E-01

Unpaired t-test, two-tailed, with alpha set to 1.00%. Each row was analysed individually, without assuming a consistent SD.

Supplementary Table 2. p-Values from unpaired t-test for the comparison of protein states WT, pS129

WT vs pS129		
Peptide	Discovery	p-Value
1-17		7.64E-01
4-17		8.40E-01
5-17		7.40E-01
5-38		3.16E-01
6-38		2.11E-01
18-38		7.07E-01
5-54		9.93E-02
18-54		1.52E-01
39-54		2.63E-02
39-56		9.20E-01
39-57		2.31E-01
39-61		6.06E-01
56-70		3.54E-02
55-94	*	6.87E-08
70-89		2.72E-01
76-89		8.53E-01
77-94		1.82E-01
77-89		9.18E-01
90-113		5.86E-01
94-113		1.28E-01
95-113		8.15E-02
114-123		1.41E-02
119-124		4.60E-01
114-124		3.39E-02
117-124		5.79E-01
133-140	*	9.80E-03
132-140		8.93E-01
135-140	*	8.09E-09

Unpaired t-test, two-tailed, with alpha set to 1.00%. Each row was analysed individually, without assuming a consistent SD.

Supplementary Table 3. p-Values from unpaired t-test for the comparison of protein states WT, WT + Ca^{2+}

WT vs (WT + Ca)		
**Peptide**	**Discovery**	**p-Value**
1-17		2.62E-01
4-17	*	6.71E-05
5-17	*	1.26E-03
5-38	*	5.86E-03
6-38	*	5.13E-05
18-38		1.62E-01
5-54	*	7.65E-04
18-54		1.48E-02
39-54	*	2.32E-03
39-56		4.31E-02
39-57		1.96E-01
39-61	*	1.52E-03
39-69	*	7.06E-04
56-70		1.98E-02
55-89	*	7.43E-05
55-94	*	2.12E-06
70-89	*	7.21E-06
76-89		5.61E-01
77-89		1.23E-01
76-94	*	3.37E-03
77-94	*	1.48E-03
90-113		3.29E-01
94-113		2.43E-01
95-113		6.22E-01
95-124		6.90E-01
114-123		1.72E-01
114-124	*	5.11E-09
117-124		1.59E-02
119-124		1.69E-01
125-131		3.25E-01
125-132	*	4.65E-04
125-134		1.32E-01
124-140		8.00E-01
125-140	*	1.28E-04
132-140	*	9.17E-03
133-140	*	2.26E-04
135-140		1.76E-01

Unpaired t-test, with alpha set to 1.00%. Each row was analysed individually, without assuming a consistent SD.

Supplementary Table 4. p-Values from unpaired t-test for the comparison of protein states D121A, D121A + Ca^{2+}

D121A vs (D121A + Ca)		
**Peptide**	**Discovery**	**p-Value**
1-17	*	6.85E-07
4-17	*	8.84E-05
5-17		3.40E-01
5-38		7.28E-02
18-38		1.49E-01
18-54		2.82E-02
39-54		4.10E-01
39-56	*	1.59E-04
39-57	*	6.97E-05
39-61	*	5.85E-03
39-69		1.72E-01
56-70		1.33E-01
55-89	*	2.28E-06
55-94	*	7.21E-09
70-89	*	9.00E-06
76-89	*	1.82E-06
76-94	*	2.87E-03
77-94	*	3.98E-07
90-113		4.61E-01
94-113		5.63E-01
95-113		6.45E-01
114-123	*	4.49E-03
114-124	*	5.33E-03
124-134	*	2.38E-05
125-134		2.45E-02
124-140	*	2.00E-05
125-140		5.55E-01
132-140		4.31E-02
133-140		6.24E-01
135-140	*	3.25E-03

Unpaired t-test, with alpha set to 1.00%. Each row was analysed individually, without assuming a consistent SD.

Supplementary Table 5. p-Values from unpaired t-test for the comparison of protein states pS129, pS129 + Ca^{2+}

pS129 vs (pS129 +Ca)		
**Peptide**	**Discovery**	**p-Value**
1-17		3.12E-01
4-17		8.27E-01
5-17		7.17E-01
5-38		4.14E-01
6-38		9.32E-01
18-38		2.78E-01
5-54		5.76E-01
18-54		4.65E-01
39-54	*	3.65E-04
39-56		4.77E-01
39-57		4.63E-01
39-61		3.76E-01
56-70		2.23E-01
55-89	*	4.38E-05
55-94	*	1.62E-04
70-89	*	7.18E-05
76-89	*	3.90E-07
77-89	*	1.97E-06
76-94	*	2.92E-03
77-94		4.58E-01
90-113		3.65E-02
94-113		8.06E-01
95-113	*	1.00E-03
114-123		4.52E-02
114-124		8.31E-01
117-124	*	8.01E-08
119-124		9.66E-01
124-140	*	1.27E-06
125-140	*	4.56E-03
133-140	*	6.54E-08
135-140		1.52E-01

Unpaired t-test, two-tailed, with alpha set to 1.00%. Each row was analysed individually, without assuming a consistent SD.

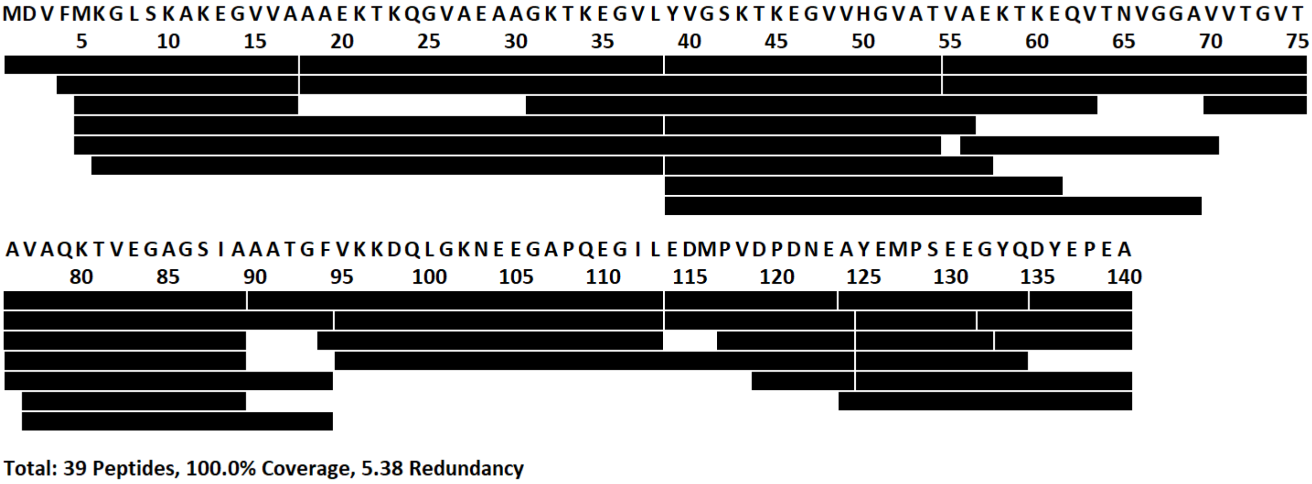

Supplementary Figure 11. Peptide coverage map of aSyn using HDX-MS
Peptide mapping of aSyn was performed on a pepsin column prior to HDX-MS experiments, yielding 100% coverage of aSyn with a high degree (5.38) of redundancy. Peptides were identified by MS-MS fragmentation with ProteinLynx Global Server (Waters).

a

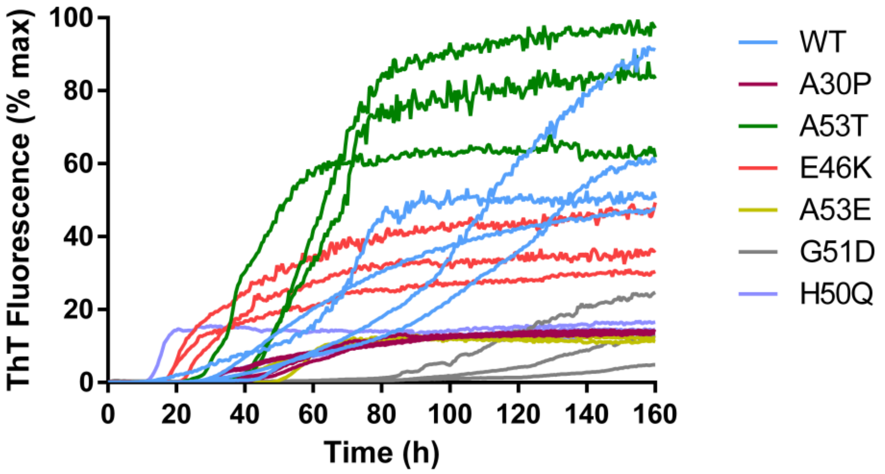

b

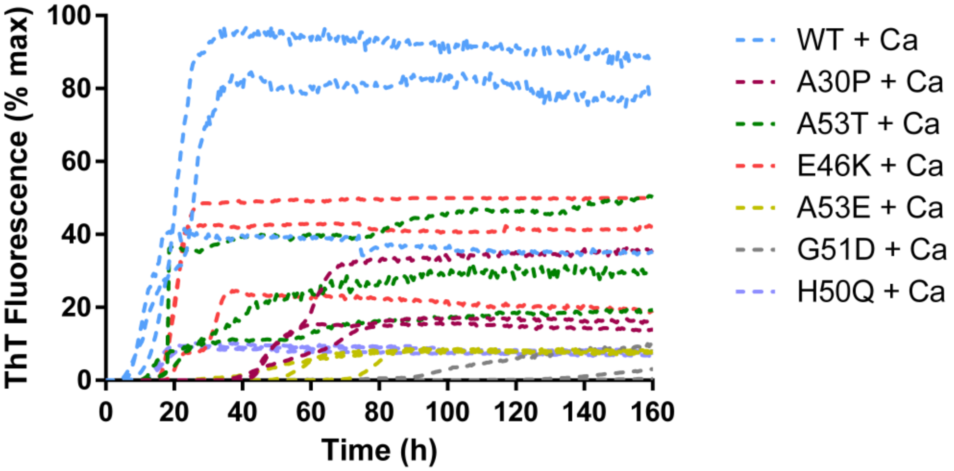

Supplementary Figure 12. ThT-based aggregation assay reveals that the aSyn familial mutants display different aggregation behaviour upon the addition of calcium Aggregation kinetics of aSyn WT and familial mutants A30P, A53T, E46K, A53E, H50Q, G51D were measured using ThT fluorescence intensity and plotted as % of maximum fluorescence. The average of at least three wells from three plate repeats are presented (A) in the absence of calcium and (B) in the presence of 2.5 mM $CaCl_2$.

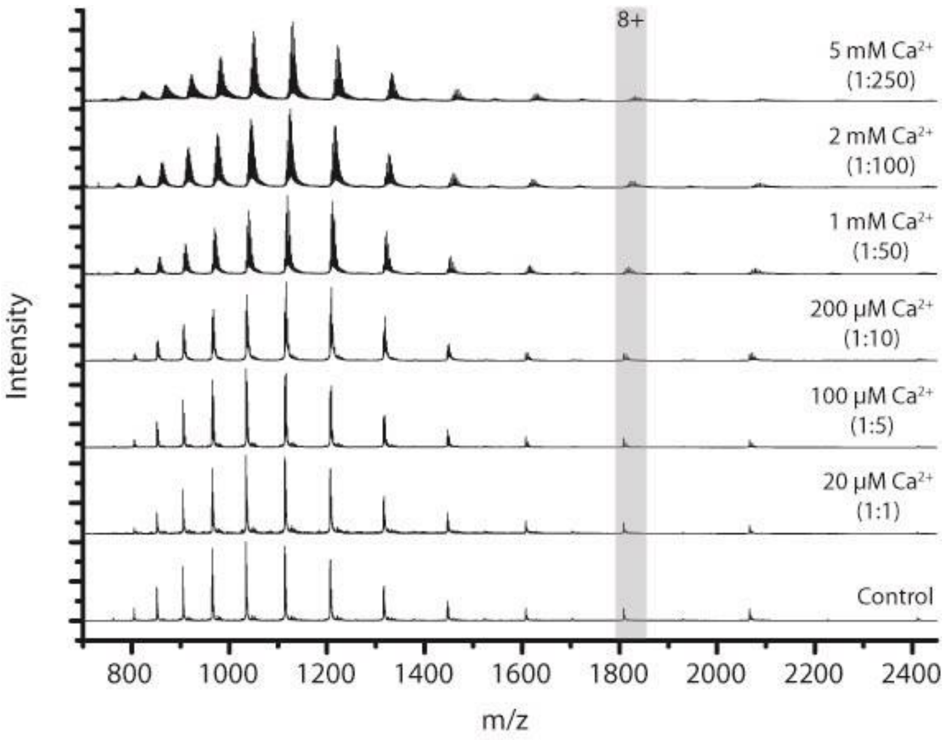

Supplementary Figure 13. Native nano ESI-MS spectra of WT aSyn with increasing concentrations of calcium The native nano ESI-MS full spectrum of WT aSyn with calcium at ratios 1:1, 1:5, 1:10, 1:50, 1:100 and 1:250.

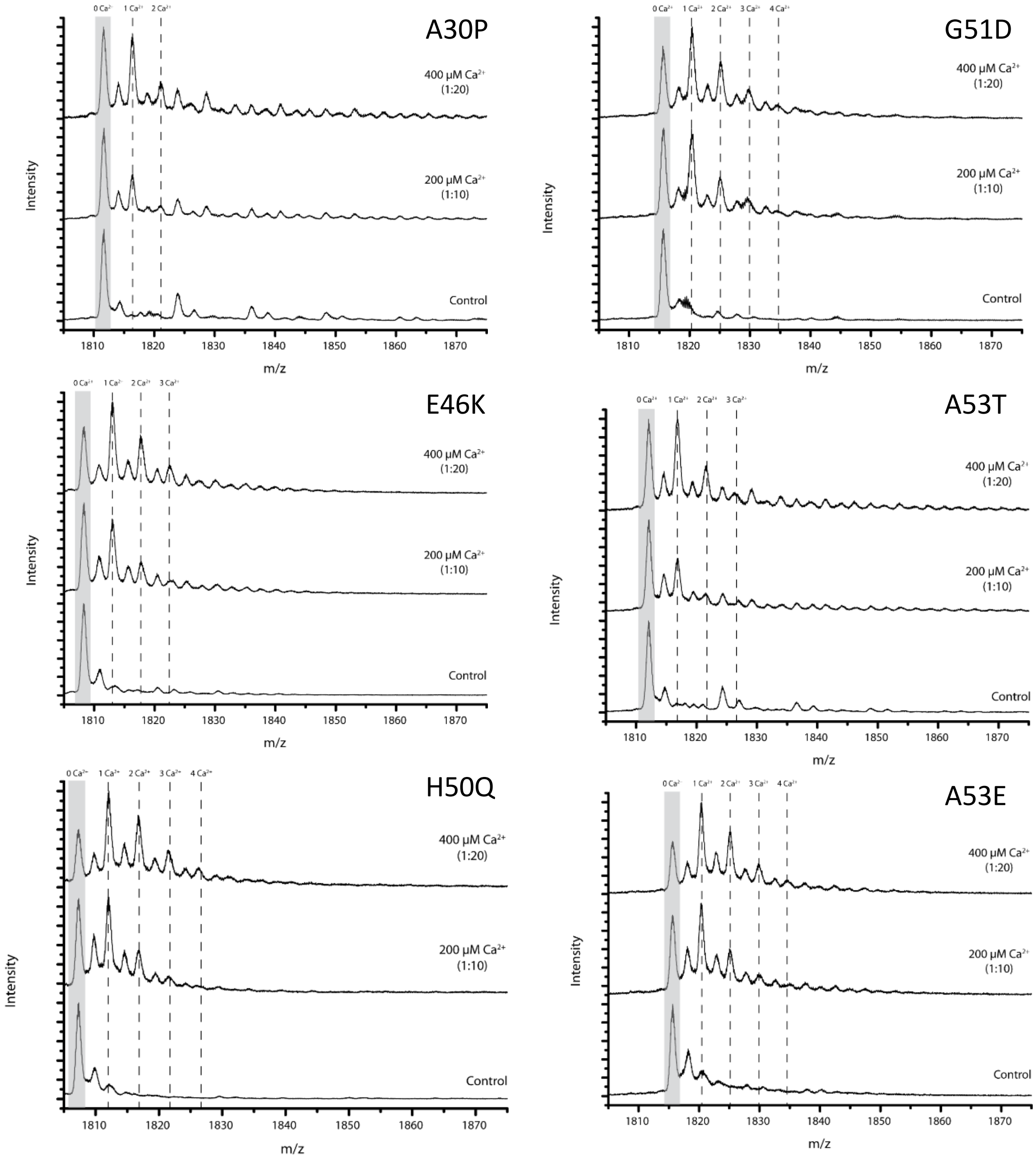

Supplementary Figure 14. Native nano ESI-MS spectra of unbound aSyn and 1:10 and 1:20 aSyn to calcium ratios for aSyn mutants.
The number of calcium ions bound to 20 µM of aSyn in 20 mM ammonium acetate at a protein to calcium ratio of 1:10 (black) and 1:50 (light grey) was determined from the mass of the different metallated species in the native nano ESI-MS spectra.

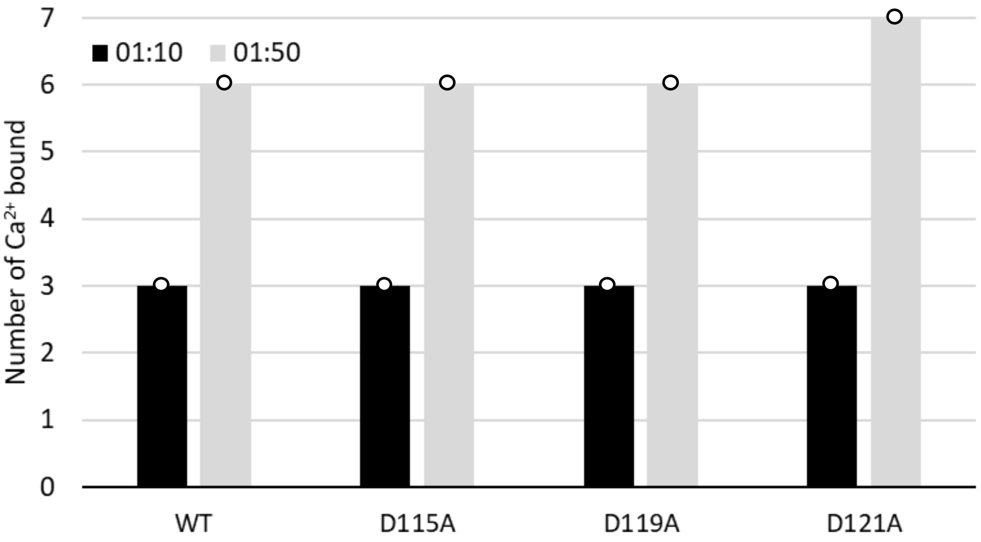

Supplementary Figure 15. Analysis of number of Ca^{2+} ions bound to the D to A aSyn mutants 1:10 and 1:50 protein to calcium ratios reveals no significant differences between the different aSyn variants

The number of calcium ions bound to 20 µM of aSyn in 20 mM ammonium acetate at a protein to calcium ratio of 1:10 (black) and 1:50 (light grey) was determined from the mass of the different metallated species in the native nano ESI-MS spectra for WT, D115A, D119A and D121A. The number of calcium ions bound in three replicates was the same for all aSyn mutants, as displayed by the circles, therefore no error is displayed.

The population intensity % (Supplementary Figure 16b) was quantified by calculating the area under the curve for each peak in the chromatograph based on drift time (Supplementary Figure 17, Supplementary Table 6 and 7). It is likely that many conformations are present in each peak, designated A-E conformation regions, as observed by the shoulders present in each peak which are not resolved (Supplementary Figure 17)

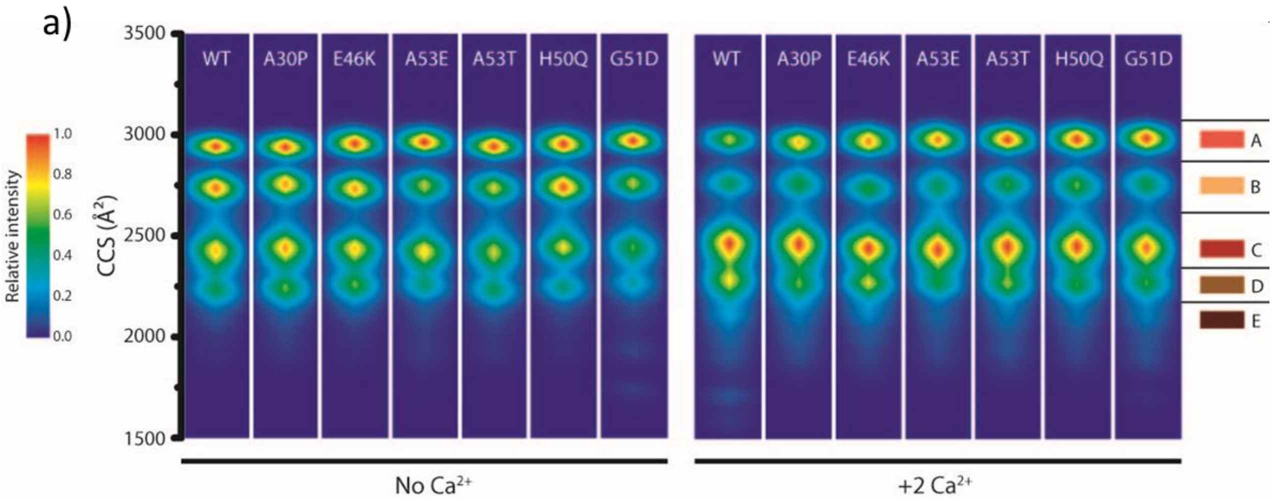

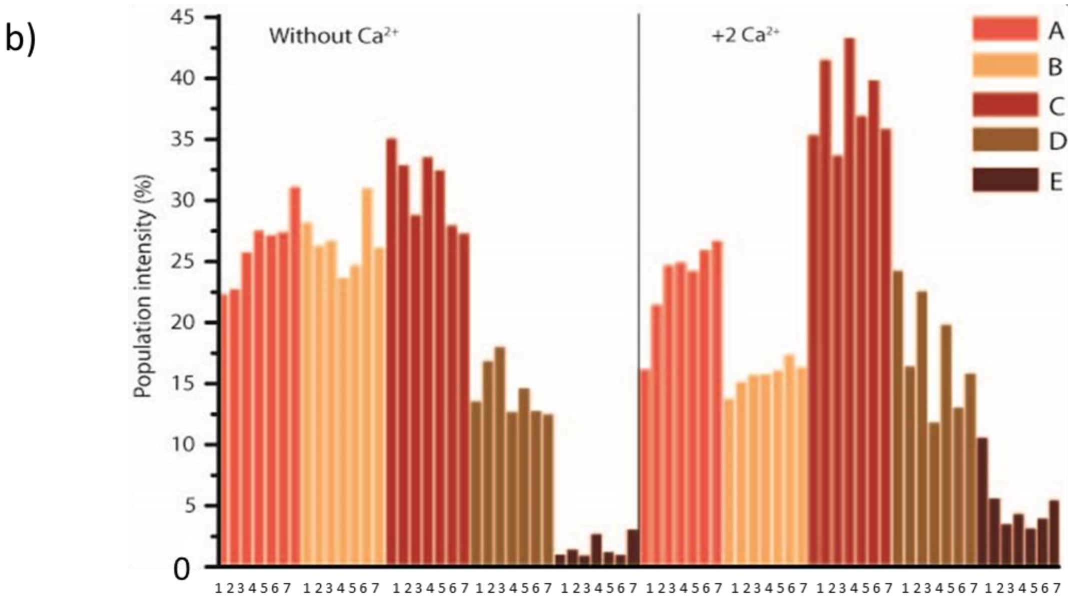

Supplementary Figure 16. Nano-ESI-IM-MS reveals several aSyn conformations in equilibria and compaction upon addition of calcium Heat maps of aSyn conformations detected for the 8+ charge state based on intensity for (a) WT aSyn and the aSyn familial mutations, A30P, E46K, A53E, A53T, H50Q and G51D in the absence (No Ca^{2+}) and presence of calcium at a 1:20 protein to calcium ratio, representing a two Ca^{2+} bound state (+2 Ca^{2+}). Red represents the most populated CCS values by intensity. Upon addition of calcium, a higher proportion of aSyn has a lower collisional cross section (CCS) value (seen in the most populated region C), representing compaction. The area is separated into regions A, B, C, D, E at the right of the heat maps to denote and quantify the population of different conformations. (b) The conformational distributions of WT and mutant aSyn (1=WT, 2=A30P, 3=E46K, 4=A53E, 5=A53T, 6=H50Q, 7=G51D) expressed as percentage were calculated from the area under peak in the ATD and are presented in the absence (Without Ca^{2+}) and presence of calcium (+2 Ca^{2+}) and also in Supplementary Tables 6 and 7 which include statistical analysis.

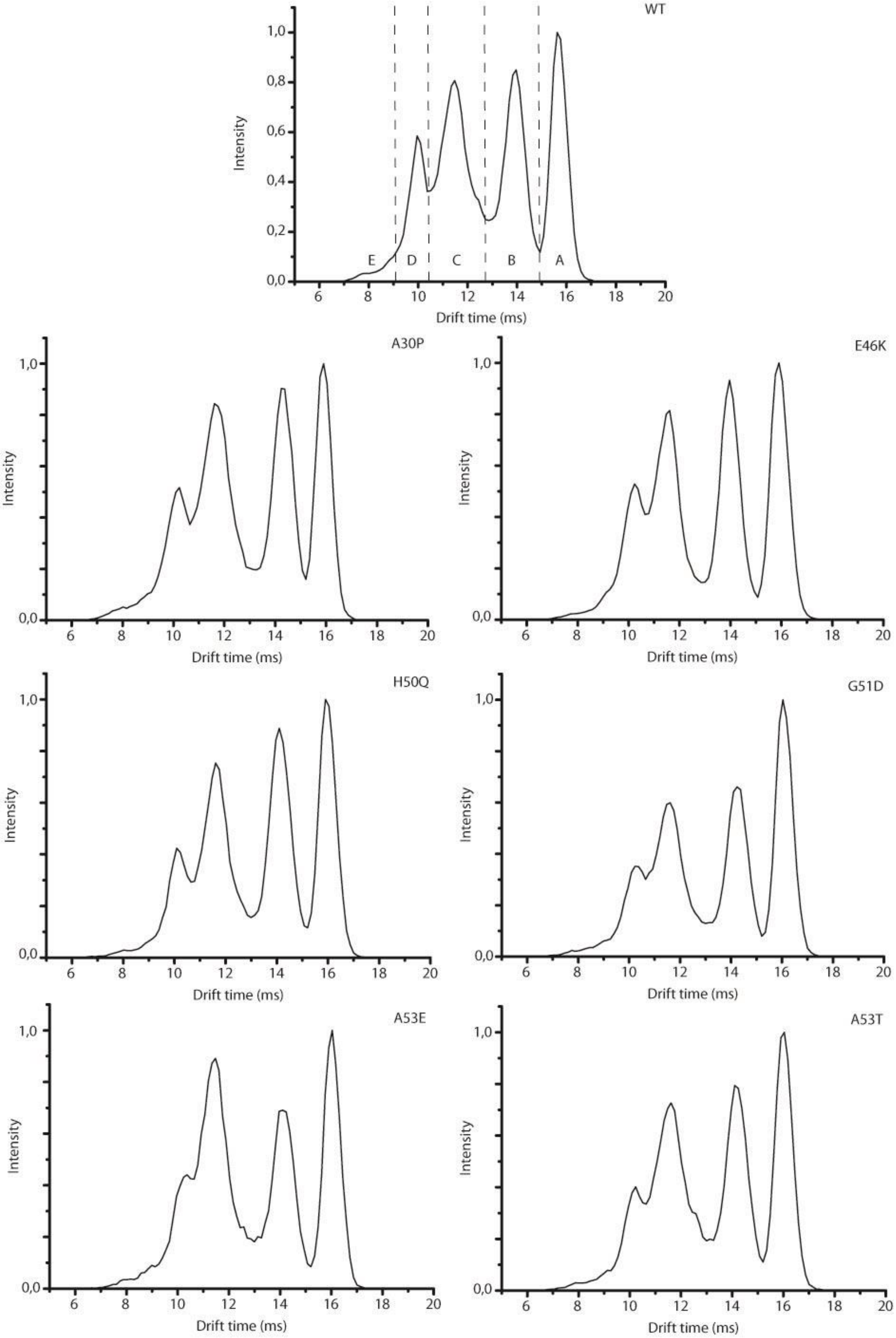

Supplementary Figure 17. Arrival time distribution (ATD) of aSyn conformations
The average percentage distribution of the conformations was determined by identifying peaks in the ATD. Each peak was designated a letter A-E dependent on ATD (represented for WT aSyn). Representative ATD are shown for WT, A30P, E46K, H50Q, G51D, A53E and A53T. Three repeat measurements were taken.

Supplementary Table 6. Average percentage of the distribution of conformations of WT aSyn and mutants determined by nano-ESI-IM-MS in the absence calcium shows small changes in percentage distribution of conformations between the aSyn and its variants

Conformation without calcium	WT av. %	A30P av. %	E46K av. %	A53E av. %	A53T av. %	H50Q av. %	G51D av. %
A	23.54 ± 0.07	21.86 ± 0.43	26.22 ± 0.28*	27.29 ± 1.32	26.57 ± 0.35*	25.96 ± 0.48	28.05 ± 1.50
B	26.80 ± 0.18	26.48 ± 0.50	27.72 ± 0.28	24.97 ± 0.25*	26.78 ± 0.10	30.47 ± 0.75	24.92 ± 1.11
C	33.09 ± 0.08	35.74 ± 0.36*	29.46 ± 0.39**	34.38 ± 1.45	32.88 ± 0.45	30.26 ± 0.79	30.49 ± 0.38*
D	15.58 ± 0.11	14.83 ± 0.79	15.74 ± 0.31	12.12 ± 0.30**	12.62 ± 0.07**	12.37 ± 0.40*	14.38 ± 1.39
E	0.98 ± 0.06	1.10 ± 0.09	0.85 ± 0.05	1.20 ± 0.30	1.10 ± 0.06	0.92 ± 0.08	2.14 ± 0.88

Conformation without calcium	WT P values	A30P P values	E46K P values	A53E P values	A53T P values	H50Q P values	G51D P values
A			0.011*		0.016		
B				0.011*			
C		0.023*	0.006**				0.027*
D				0.005**	0.0002**	0.016*	
E							

Supplementary Table 7. Average percentage of the distribution of conformations of WT and aSyn mutants determined by nano-ESI-IM-MS in the presence of calcium shows significant differences upon the addition of calcium to aSyn and its variants

Conformation with 2 Ca^{2+} bound	WT av. %	A30P av. %	E46K av. %	A53E av. %	A53T av. %	H50Q av. %	G51D av. %
A	21.53 ± 0.69	22.79 ± 0.94	25.82 ± 0.38*	24.18 ± 0.93	25.40 ± 0.18*	24.86 ± 0.98	25.00 ± 0.96
B	14.73 ± 0.30	19.96 ± 0.42**	17.54 ± 0.32**	15.39 ± 0.17	18.66 ± 0.36**	17.74 ± 0.35**	16.06 ± 0.29
C	37.85 ± 0.36	37.57 ± 0.64	32.74 ± 0.08**	39.04 ± 0.95	38.66 ± 0.91	37.74 ± 0.34	36.16 ± 0.40
D	22.83 ± 0.42	17.57 ± 0.93*	21.58 ± 0.32	18.93 ± 0.79*	15.96 ± 0.43**	18.06 ± 1.12	20.10 ± 0.92
E	3.07 ± 0.21	2.10 ± 0.17*	2.32 ± 0.14	2.45 ± 0.11	1.84 ± 0.10*	2.16 ± 0.09	2.68 ± 0.12

Conformation with 2 Ca^{2+} bound	WT P values	A30P P values	E46K P values	A53E P values	A53T P values	H50Q P values	G51D P values
A			0.02*		0.04*		
B		0.002**	0.006**		0.003**	0.006**	
C			0.005**				
D		0.03*		0.04*	0.0007**		
E		0.04*			0.02*		

*p<0.05
**p<0.01
p values calculated using an unpaired T-test without assuming equal variance from the average of three replicates of mutant aSyn compared to average of three replicates of WT aSyn

A30P

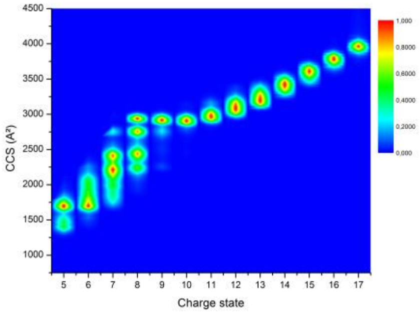

E46K

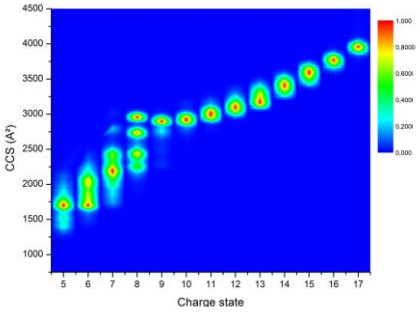

H50Q

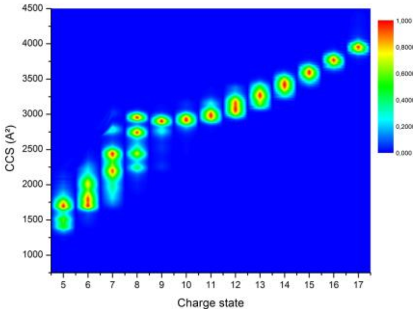

G51D

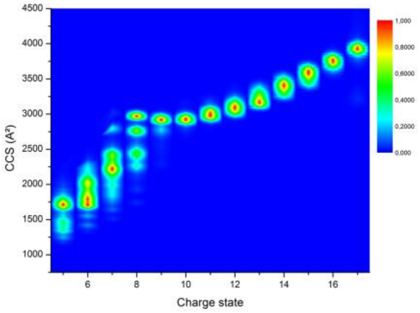

A53T

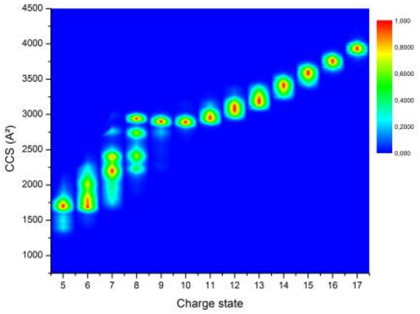

A53E

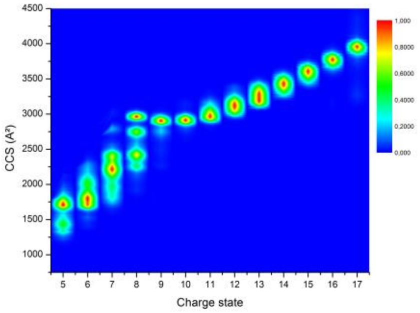

WT

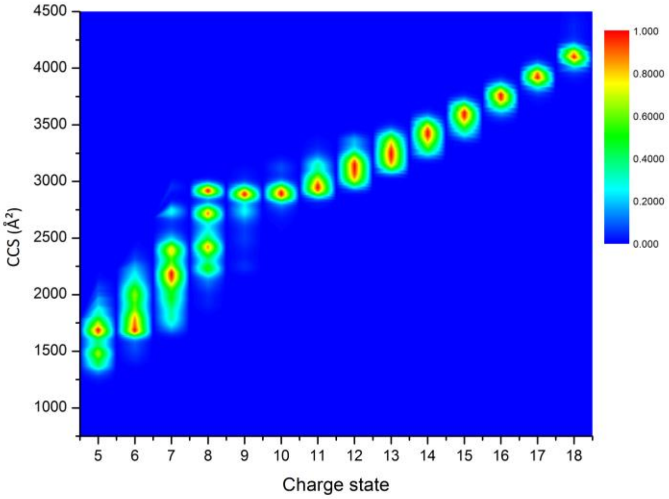

Supplementary Figure 18. CCS values of aSyn WT and familial mutants show multiple coexisting conformations and extensions at higher charge states
Nano-ESI-IM-MS spectra of WT aSyn and aSyn familial mutants at all charge states detected (20 µM of aSyn in 50 mM ammonium acetate). Multiple defined conformations can be observed at charge states +9 and below. At the +10 charge state and above, charge repulsion leads to an extended, chain-like conformation as shown by the increasing CCS values. Representative data shown, three injections performed.

A30P

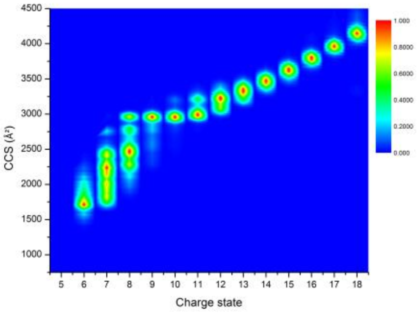

E46K

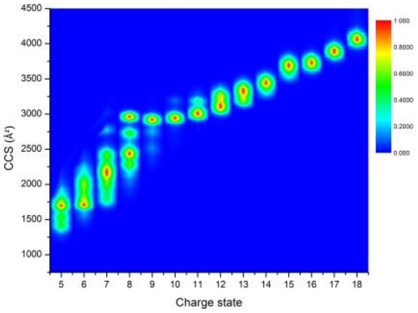

H50Q

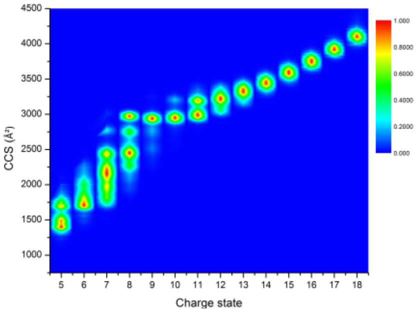

G51D

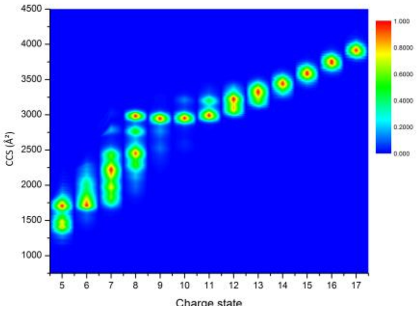

A53T

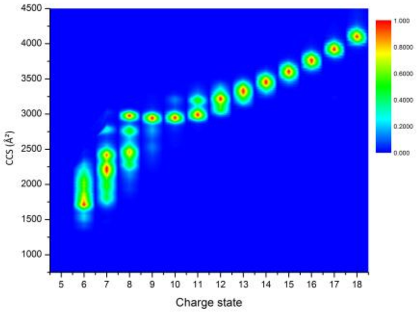

A53E

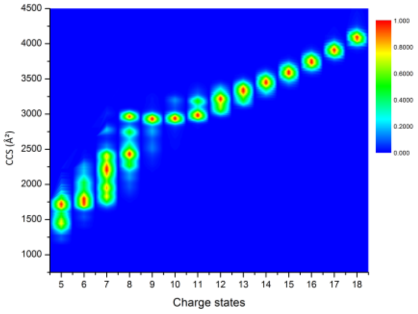

Supplementary Figure 19. CCS values of aSyn WT and familial mutants bound to two calcium ions show multiple coexisting conformations Nano-ESI-IM-MS spectra of WT aSyn and aSyn familial mutants with two calcium ions bound at all charge states detected (20 µM of aSyn with 400 µM $CaCl_2$ in 50 mM ammonium acetate). Compacted conformations are observed at lower charge states (+5 - +9). Multiple defined conformations can be observed at charge states +9 and below. At the +10 charge state and above, charge repulsion leads to an extended, chain-like conformation as shown by the increasing CCS values. For A30P and A53T the intensity of the +5 charge state was too low to obtain a drift time profile. Representative data shown, three injections performed.

Supplementary Note 2

Discussion of nano-ESI-IM-MS

Techniques such as NMR and HDX-MS measure the average of the ensemble of all conformations in the sample. Native nano-ESI IM-MS can instead resolve heterogeneous structures in the ensemble, which may permit us to determine whether there is a difference in the distribution of conformations between different aSyn variants. The 8+ charge state was chosen because it is one of the intermediate charge states which resembles conformations present in solution elucidated by NMR[1]; this state has multiple, clearly defined CCS distributions which reflect physiologically relevant states. The CCS values at 8+ were subdivided into five regions labelled A-E, regions A-D contained the most populated conformations, with region E containing the least abundant and most compact conformations (Supplementary Figure 16, 17). The presence of shoulders on some of the peaks shown in the chromatograph of arrival time distribution (ATD) suggest multiple conformations are present within each peak (Supplementary Figure 17). As the charge state increases, the aSyn conformations become more extended due to higher Coulombic repulsion (Supplementary Figure 18).

The familial mutations also display five co-existing conformational distributions, again with regions A-D being the most populated (Supplementary Figure 16, 17). To determine differences in conformation distribution, we analysed the data by both intensity (Supplementary Figure 16a) and calculation of the area under each peak in the ATD, displayed as a percentage of the total population (Supplementary Figure 16b, 17). No significant differences in the distribution of the ensemble of conformations between WT and mutant aSyn were observed, there are however slight differences in the distribution of conformations between the different aSyn variants (Supplementary Figure 16, Supplementary Table 6). Furthermore, there were no clear differences in the distribution of conformations at other charge states between the aSyn variants (Supplementary Figure 18, 19).

Upon addition of calcium, compacted conformations are favoured in all aSyn variants, particularly conformation C, as indicated by more populated low CCS values (Supplementary Figure 16a, $+2Ca^{2+}$). There are currently two possible explanations for the observed compaction of aSyn upon calcium binding, either the C-terminus collapses due to charge neutralisation removing the electrostatic repulsion, which has also been observed for Mn^{2+} and Co^{2+} binding [2] or that the gas phase induces compaction upon specific divalent cation binding which leads to charge neutralisation and electrostatic interactions being favoured, which may not occur in solution [3]. However, these findings do indicate that monomeric aSyn can manifest in a variety of conformations. They further indicate that calcium significantly skews the conformational ensemble of the different aSyn variants. However, the technique is though not yet capable to resolve clear structural differences between the aSyn variants and WT aSyn and it is currently not clear how these distributions of conformations in the gas phase relate to solution conditions.

Supplementary Table 8. p-Values from unpaired t-test for the comparison of protein states WT, A53T

WT vs A53T		
**Peptide**	**Discovery**	**p-Value**
1-17		2.65E-01
4-17	*	4.85E-03
5-17		1.69E-01
5-38		9.81E-01
6-38		5.98E-02
56-70		1.01E-02
55-89		5.57E-01
55-94		5.28E-01
70-89		3.82E-02
76-89		9.22E-01
77-89		9.96E-01
76-94		2.34E-01
77-94		2.05E-01
90-113		6.13E-01
94-113		8.20E-01
95-113		5.89E-01
95-124		4.43E-01
114-123		9.79E-01
114-124		7.53E-01
117-124		1.51E-02
119-124		4.26E-01
125-131		1.72E-01
125-132		6.45E-01
125-134		9.67E-01
125-140		1.37E-01
132-140		3.85E-01
133-140		6.38E-01
135-140		9.85E-01

Unpaired t-test, two-tailed, with alpha set to 1.00%. Each row was analysed individually, without assuming a consistent SD.

Supplementary Table 9. p-Values from unpaired t-test for the comparison of protein states WT, A53E

WT vs A53E		
**Peptide**	**Discovery**	**p-Value**
1-17		9.85E-01
4-17		2.17E-01
5-17		8.19E-01
5-38		3.71E-01
6-38		2.63E-01
55-89		1.99E-01
55-94		6.64E-02
70-89		9.41E-01
76-89		3.91E-01
77-89		4.50E-01
76-94		7.82E-01
77-94		2.72E-01
90-113		8.59E-02
94-113		2.96E-01
95-113		7.64E-02
95-124		3.98E-01
114-123		8.53E-02
114-124		7.88E-01
117-124		1.46E-01
119-124		3.84E-01
125-131		1.56E-01
125-132		3.60E-01
125-134		4.78E-01
125-140		2.24E-01
132-140		4.79E-01
133-140		4.29E-01
135-140		7.07E-01

Unpaired t-test, two-tailed, with alpha set to 1.00%. Each row was analysed individually, without assuming a consistent SD.

Supplementary Table 10. p-Values from unpaired t-test for the comparison of protein states A53T, A53E

A53T vs A53E		
**Peptide**	**Discovery**	**p-Value**
1-17		1.62E-01
4-17		6.06E-02
5-17		2.20E-01
5-38		4.03E-01
6-38		1.89E-01
55-89		5.34E-01
55-94		1.35E-01
70-89		2.40E-01
76-89		1.12E-01
77-89		3.87E-02
76-94		4.11E-01
77-94		2.27E-01
90-113		2.56E-01
94-113		1.24E-01
95-113		1.09E-01
95-124		7.63E-01
114-123		3.46E-02
114-124		6.83E-01
117-124		1.95E-01
119-124		1.96E-01
125-131		3.28E-01
125-132		9.03E-01
125-134		8.77E-01
125-140		6.05E-01
132-140		2.62E-01
133-140		5.99E-01
135-140		5.49E-01

Unpaired t-test, two-tailed, with alpha set to 1.00%. Each row was analysed individually, without assuming a consistent SD.

Supplementary Table 11. p-Values from unpaired t-test for the comparison of protein states A53T, A53T + Ca^{2+}

A53T vs (A53T + Ca)		
**Peptide**	**Discovery**	**p-Value**
1-17		2.07E-01
4-17		3.51E-01
5-17		1.99E-02
5-38-	*	4.67E-04
6-38	*	1.45E-04
18-38		5.39E-01
5-54	*	1.24E-03
39-54		2.44E-01
39-57	*	3.81E-07
39-61		8.35E-01
39-68	*	2.73E-03
55-89		1.45E-01
55-94		5.12E-01
70-89		4.68E-01
76-89		3.25E-01
77-89		4.61E-01
76-94		1.04E-01
77-94	*	4.25E-04
90-113		6.99E-01
94-133		2.95E-01
95-113		5.22E-01
95-124	*	5.32E-03
114-123		3.35E-01
114-124	*	2.43E-05
117-124		5.74E-01
119-124		5.66E-01
125-131	*	5.85E-08
125-132		8.95E-02
124-134	*	2.59E-03
125-134		6.63E-01
125-140	*	2.55E-04
132-140	*	8.13E-05
133-140	*	4.24E-05
135-140	*	1.04E-07

Unpaired t-test, two-tailed, with alpha set to 1.00%. Each row was analysed individually, without assuming a consistent SD.

Supplementary Table 12. p-Values from unpaired t-test for the comparison of protein states A53E, A53E + Ca^{2+}

A53E vs (A53E + Ca)		
**Peptide**	**Discovery**	**p-Value**
1-17		4.94E-01
4-17		7.63E-01
5-17		9.99E-01
5-38		7.21E-01
18-38		3.22E-01
39-54		3.79E-01
39-56		9.49E-01
39-69		4.80E-01
55-89	*	7.49E-04
55-94	*	2.85E-04
70-89		3.80E-01
76-89		1.35E-01
77-89		8.34E-02
76-94		3.15E-02
77-94		2.63E-01
90-113		8.72E-01
94-113		7.00E-01
95-113		4.05E-01
95-124		4.47E-01
114-123	*	1.55E-05
114-124		7.32E-02
117-124	*	2.55E-03
119-124		3.70E-01
124-134		5.06E-01
125-134	*	8.30E-03
124-140	*	7.67E-08
125-140		8.85E-01
132-140		4.84E-01
133-140	*	3.94E-08
135-140		5.57E-01

Unpaired t-test, two-tailed, with alpha set to 1.00%. Each row was analysed individually, without assuming a consistent SD.

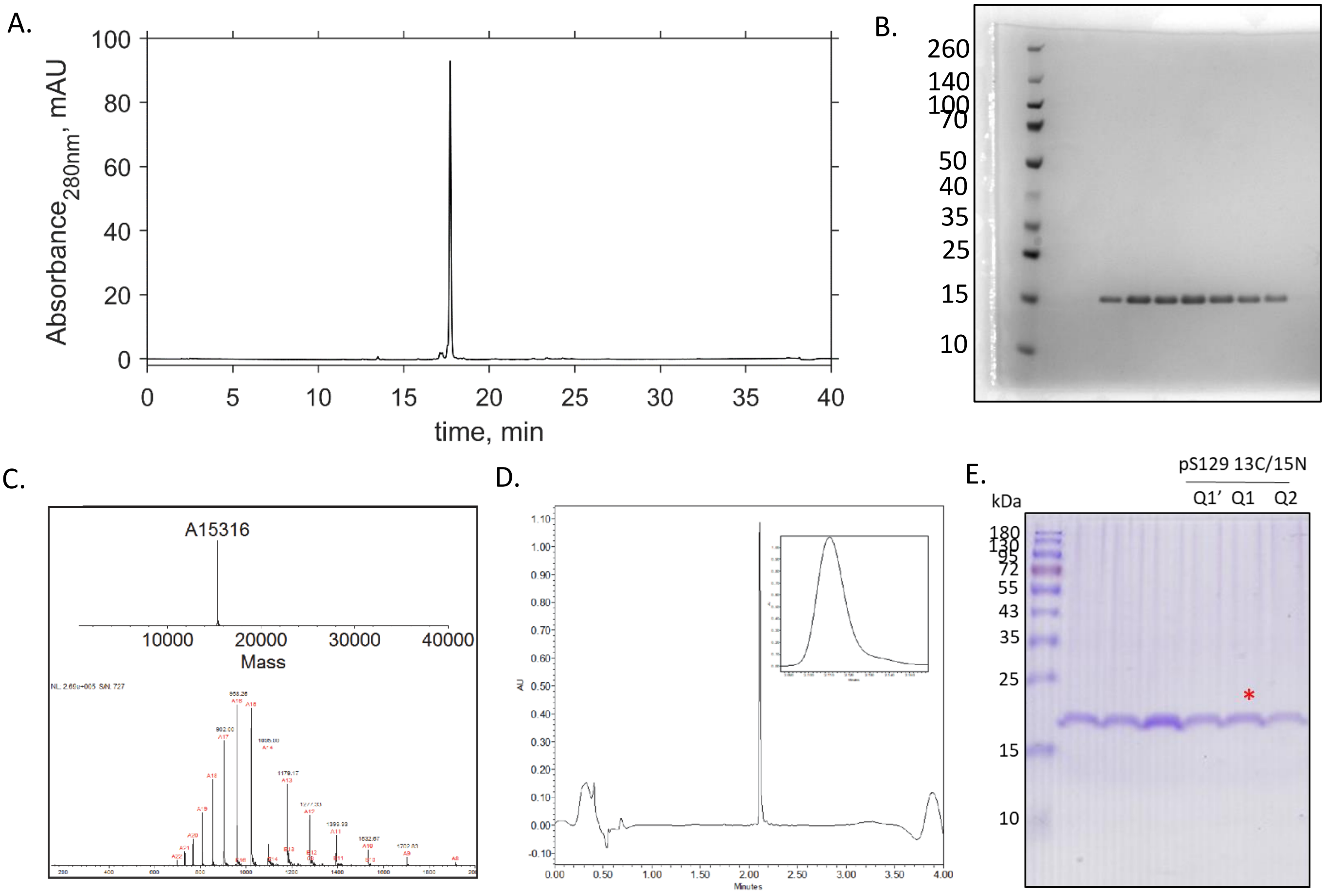

Supplementary Figure 20. Analysis of WT and $^{13}C/^{15}N$ pS129 aSyn purity and percentage phosphorylation

(A) Representative chromatograph of WT aSyn analysed by analytical reverse phase (RP)-HPLC, 50 µL of aSyn was injected onto a Discovery Bio Wide Pore C18-5 column and eluted using a gradient of 5% acetonitrile + 0.1% acetic acid to 95% acetonitrile + 0.1% acetic acid with H_2O + 0.1% acetic acid over 40 minutes at 1 ml/min. Percentage purity of aSyn was 93.9% based on absorbance at 280 nm and ranged from 89-96% for multiple purification runs. (B) Coomassie blue staining of SDS-PAGE gel of WT aSyn at ~15 kDa. (C) Phosphorylation of $^{13}C/^{15}N$ labelled aSyn at S129 was confirmed by mass spectrometry (expected mass 15.333 kDa). (D) Ultra performance liquid chromatography (UPLC) and (E) Coomassie blue staining of SDS-PAGE gel was used to determine aSyn purity, the red star shows the fraction used in experiments. The labelling percentage of S129 was 99.89%.

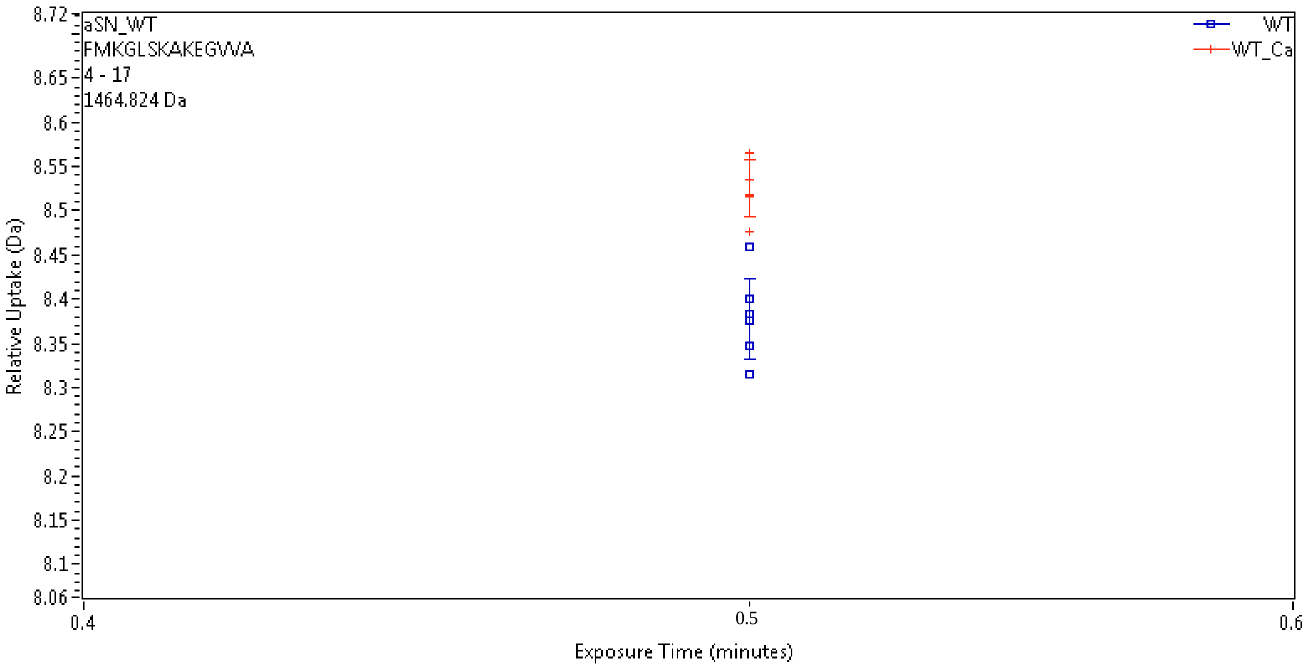

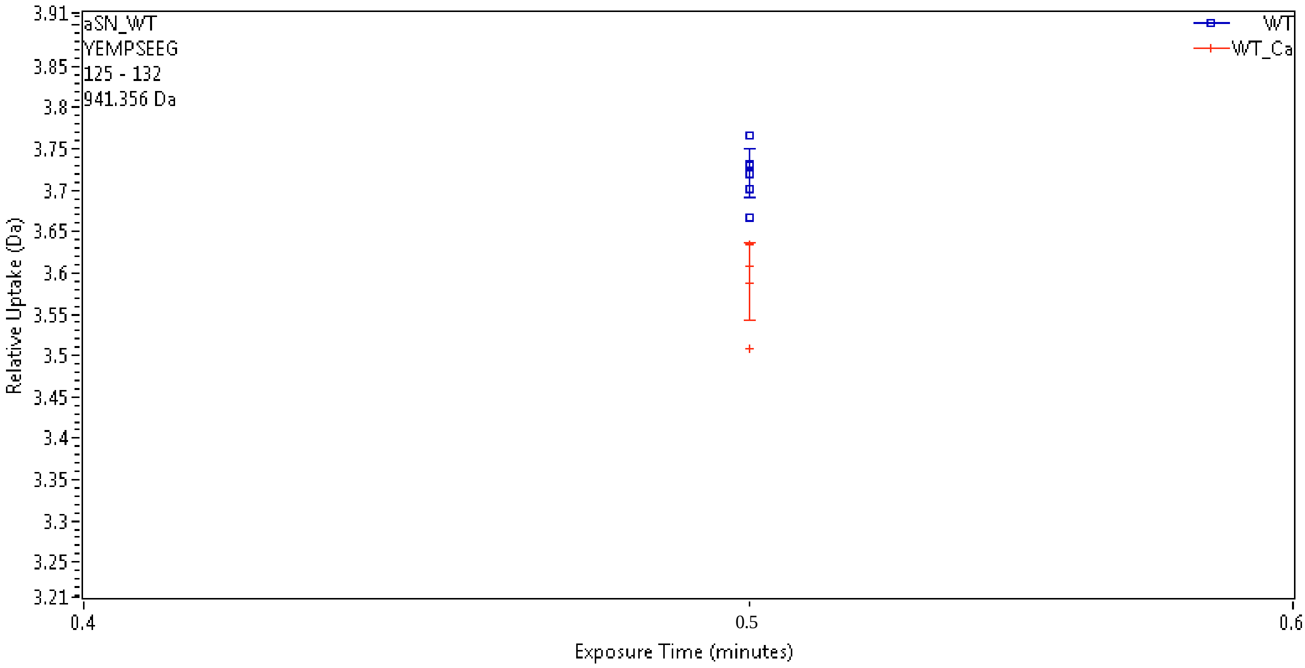

Supplementary Figure 21. Deuterium uptake plots, measured in Dalton (Da), for representative peptides containing aar 4-17 (N-terminus) and aar 125-132 (C-terminus) of WT aSyn in the presence (red) and absence (blue) of calcium Six replicates were collected per condition (+/- calcium) for a 30 s labelling timepoint. Error indicates 1 s.d. No correction was made for back-exchange. Uptake data for all the peptides were collected and further analysed to form Figures 3 and 5.

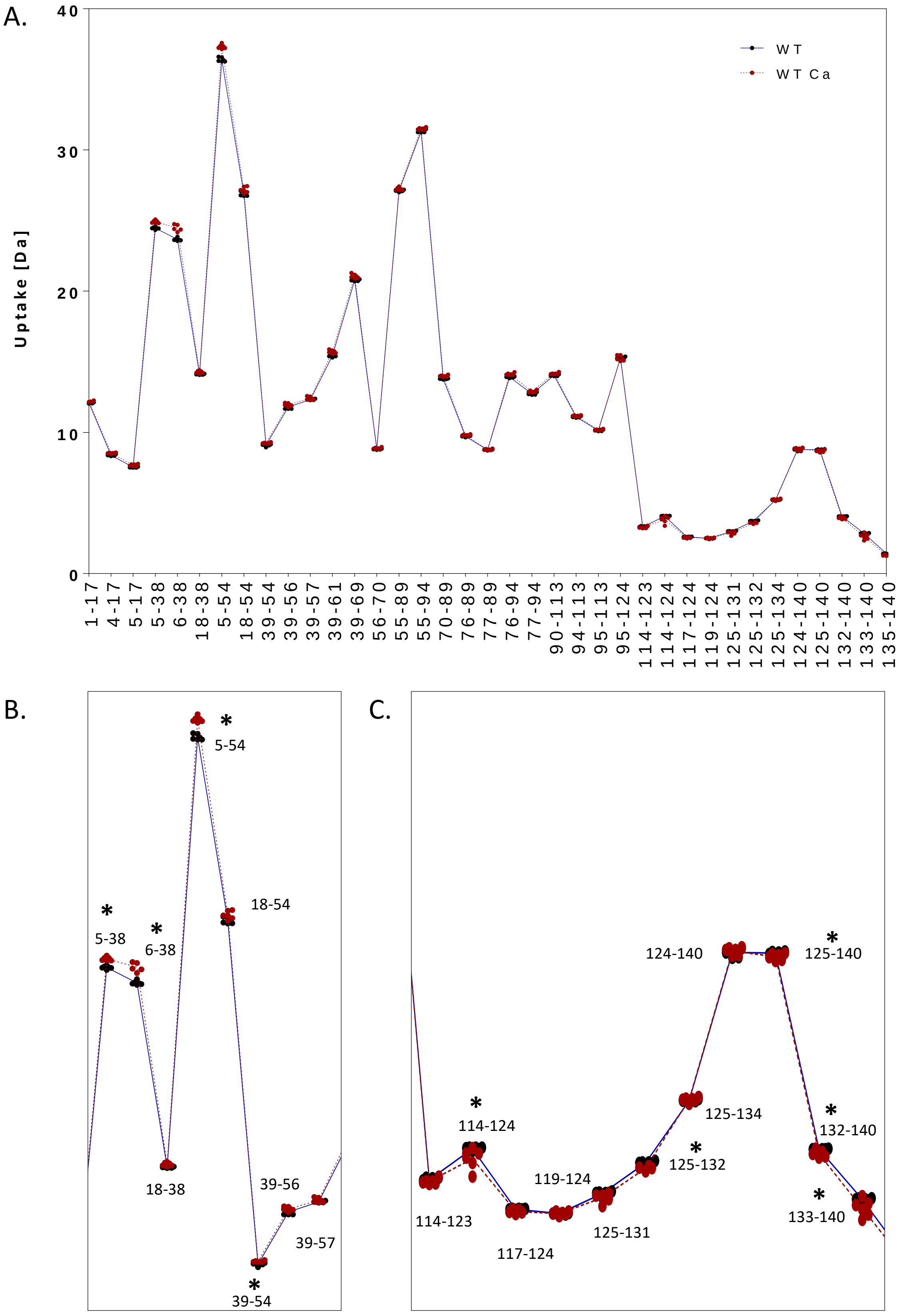

Supplementary Figure 22. Deuterium uptake plots, measured in Dalton (Da), for WT aSyn in the presence (red) and absence (blue) of calcium, showing the individual replicates for each measurement. Data correspond to manuscript main figure 3c and 6d. **A.** Deuterium uptake throughout the sequence, **B.** Zoomed-in section of the N-terminus, highlighting peptides with significant differences (5-38, 6-38, 5-54, 39-54), C. Zoomed in section of the C-terminus, highlighting peptides with significant differences (114-124, 125-132, 125-140, 132-140, 133-140).

A.

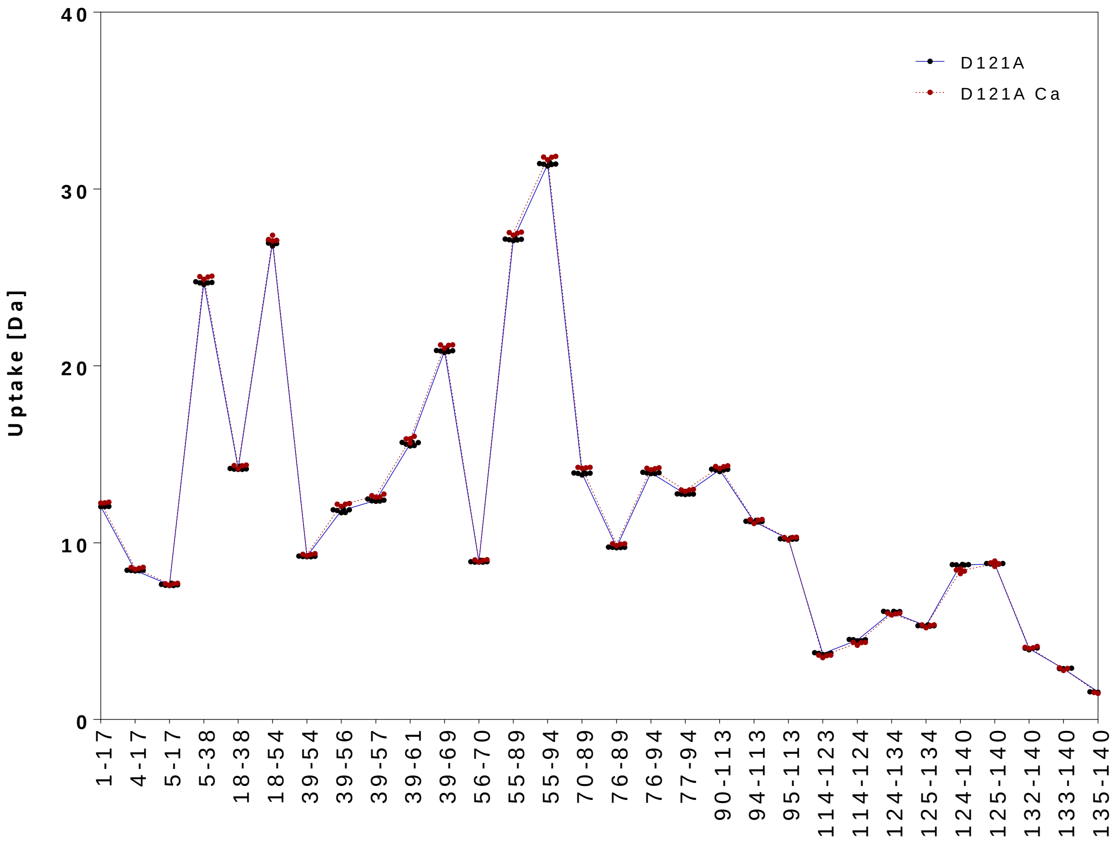

B.

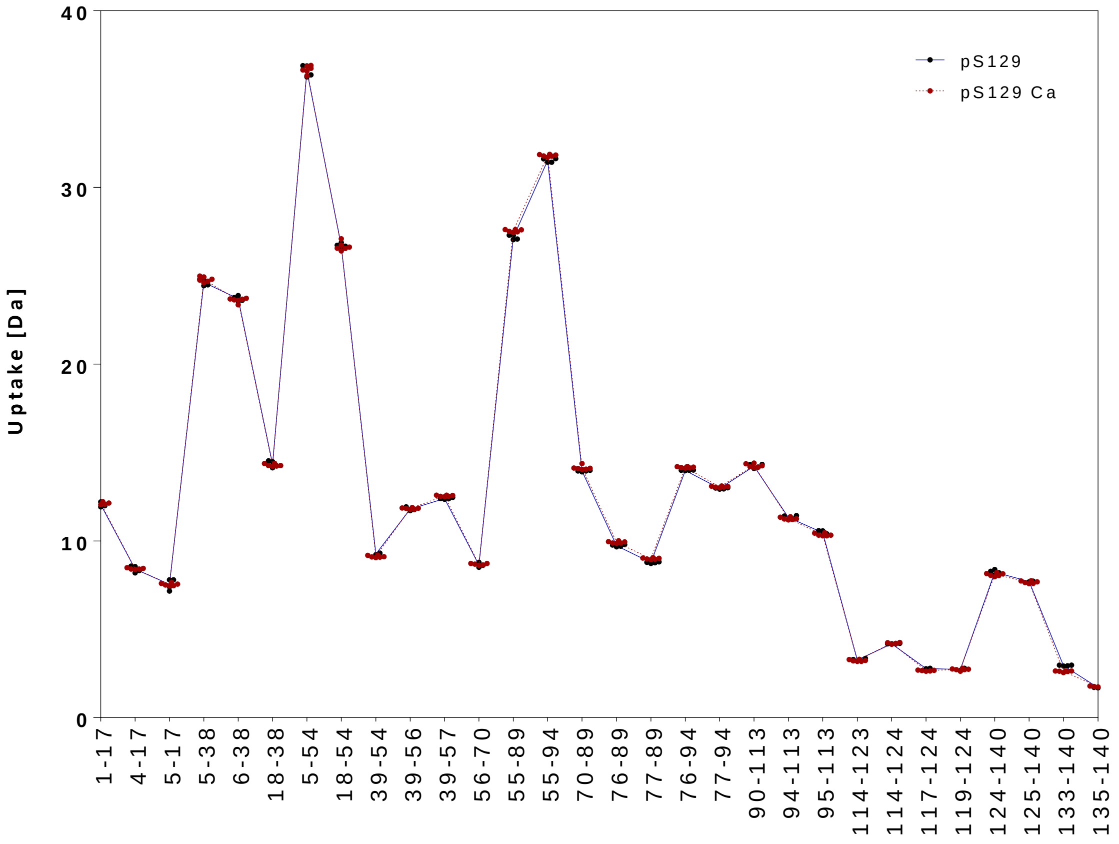

Supplementary Figure 23. Deuterium uptake plots, measured in Dalton (Da), for A. D121A aSyn in the presence (red) and absence (blue) of calcium, and B. pS129 aSyn in the presence (red) and absence (blue) of calcium, showing the individual replicates for each measurement. Data correspond to manuscript main figures 3d and 3e.

A.

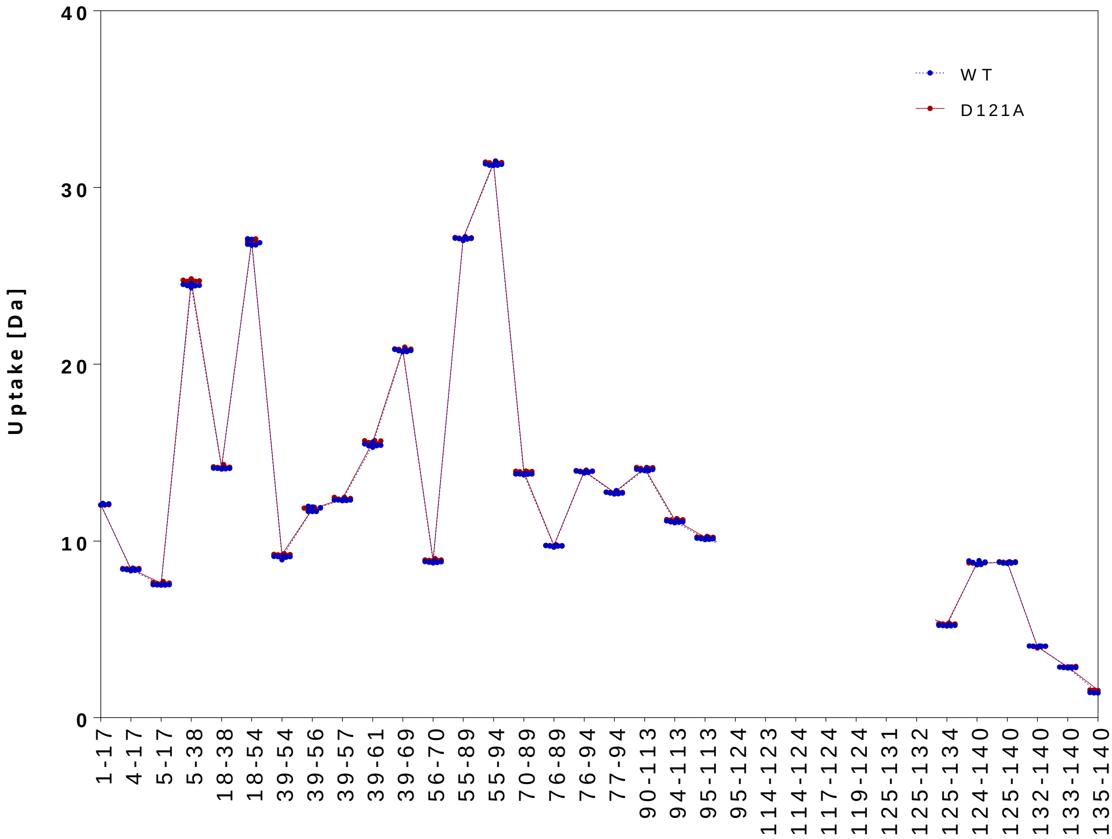

B.

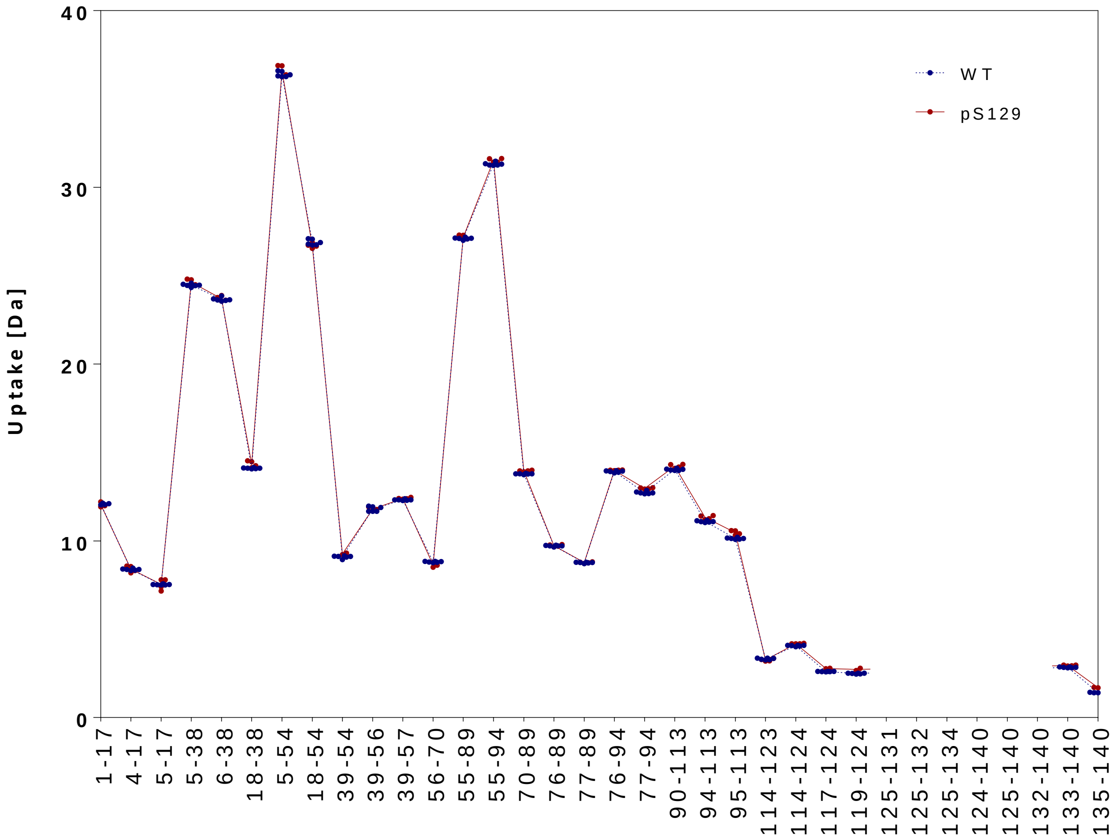

Supplementary Figure 24. Deuterium uptake plots, measured in Dalton (Da), for A. WT aSyn (blue) and D121A aSyn (red), and B. WT aSyn (blue) and pS129 aSyn (red), showing the individual replicates for each measurement. Data correspond to manuscript main figures 3a and 3b.

A.

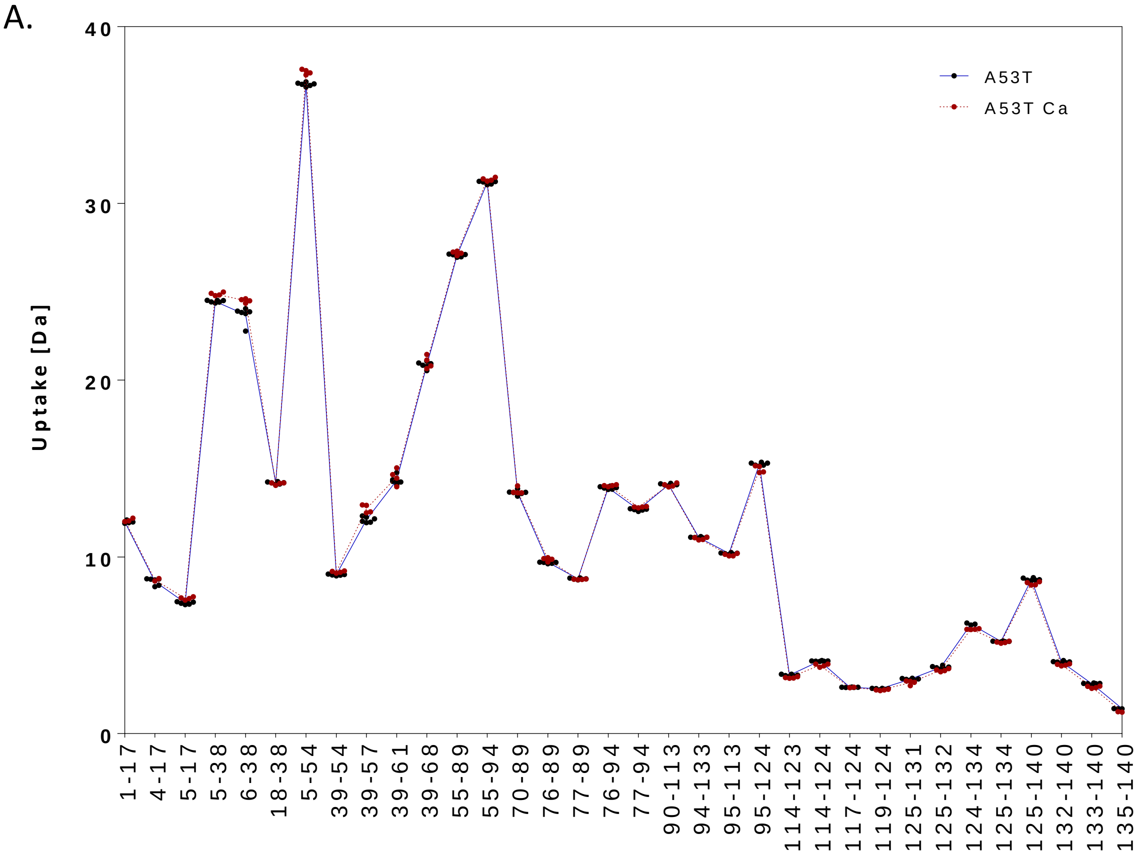

B.

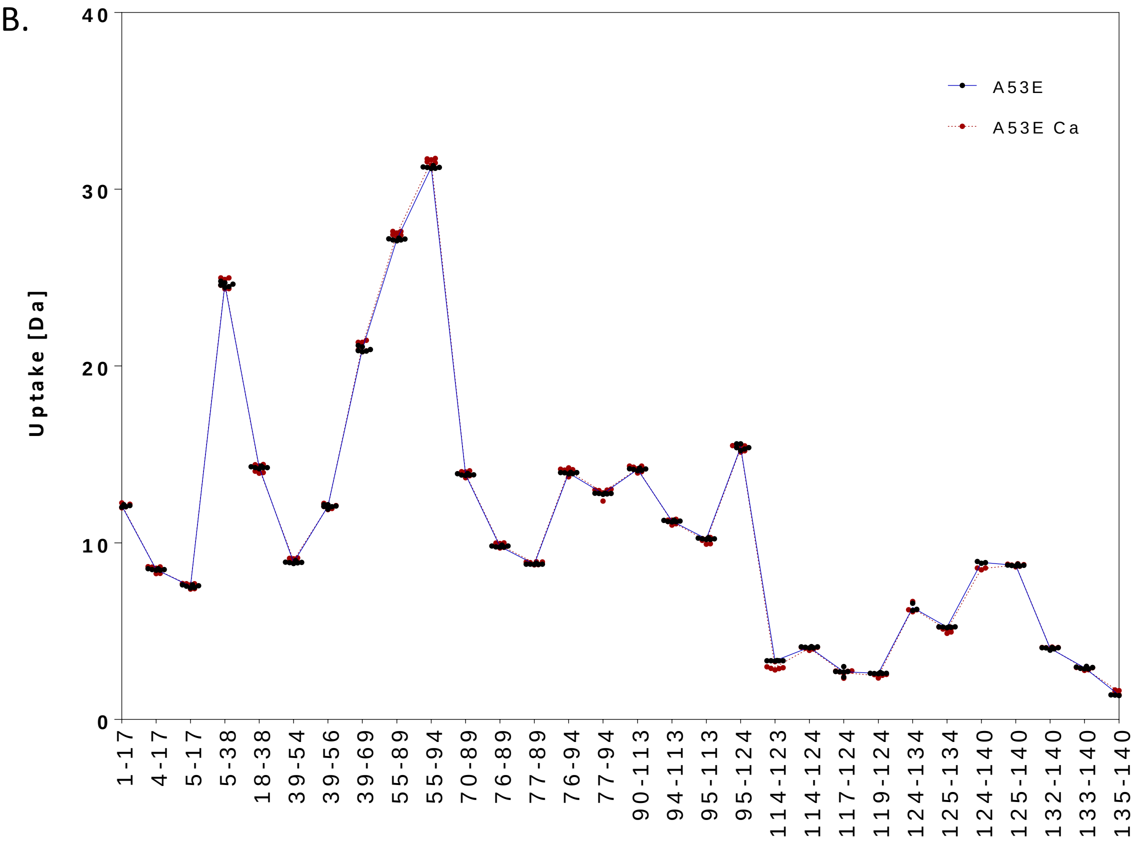

Supplementary Figure 25. Deuterium uptake plots, measured in Dalton (Da), for A. A53T aSyn in the presence (red) and absence (blue) of calcium, and B. A53E aSyn in the presence (red) and absence (blue) of calcium, showing the individual replicates for each measurement. Data correspond to manuscript main figures 6e and 6f.

A.

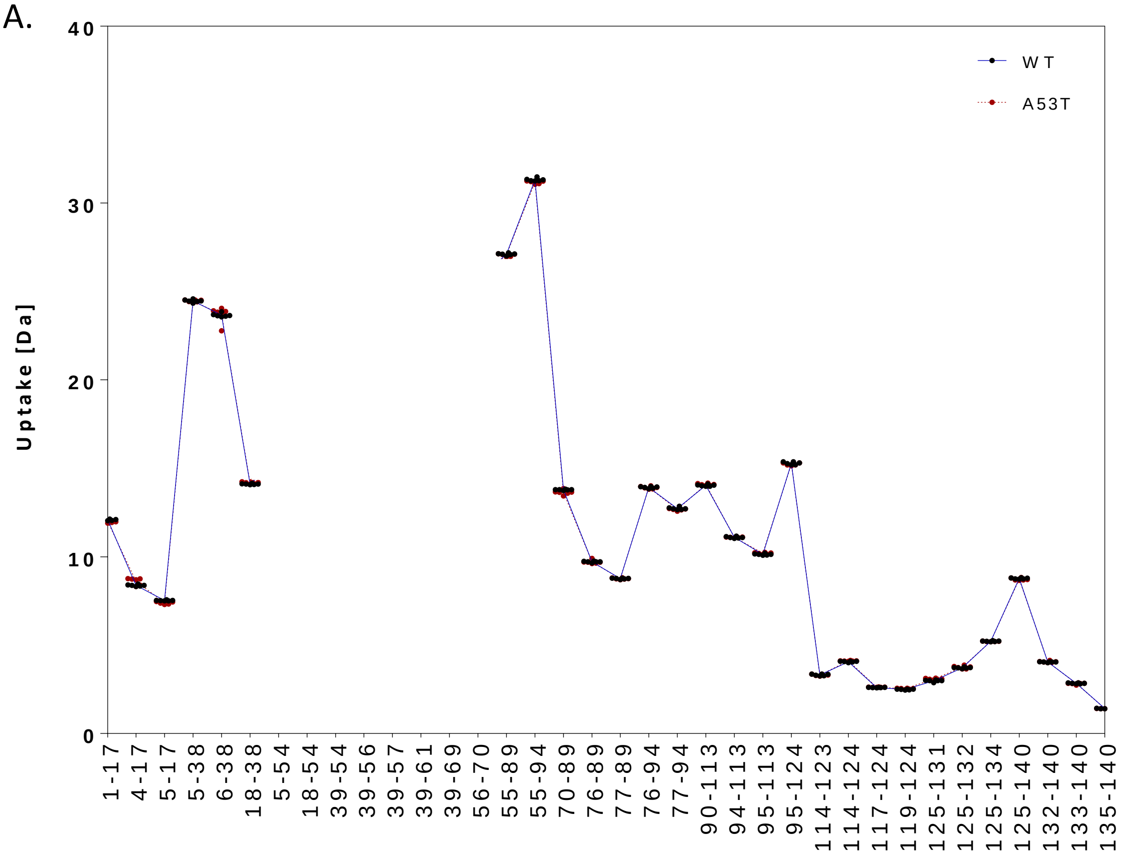

B.

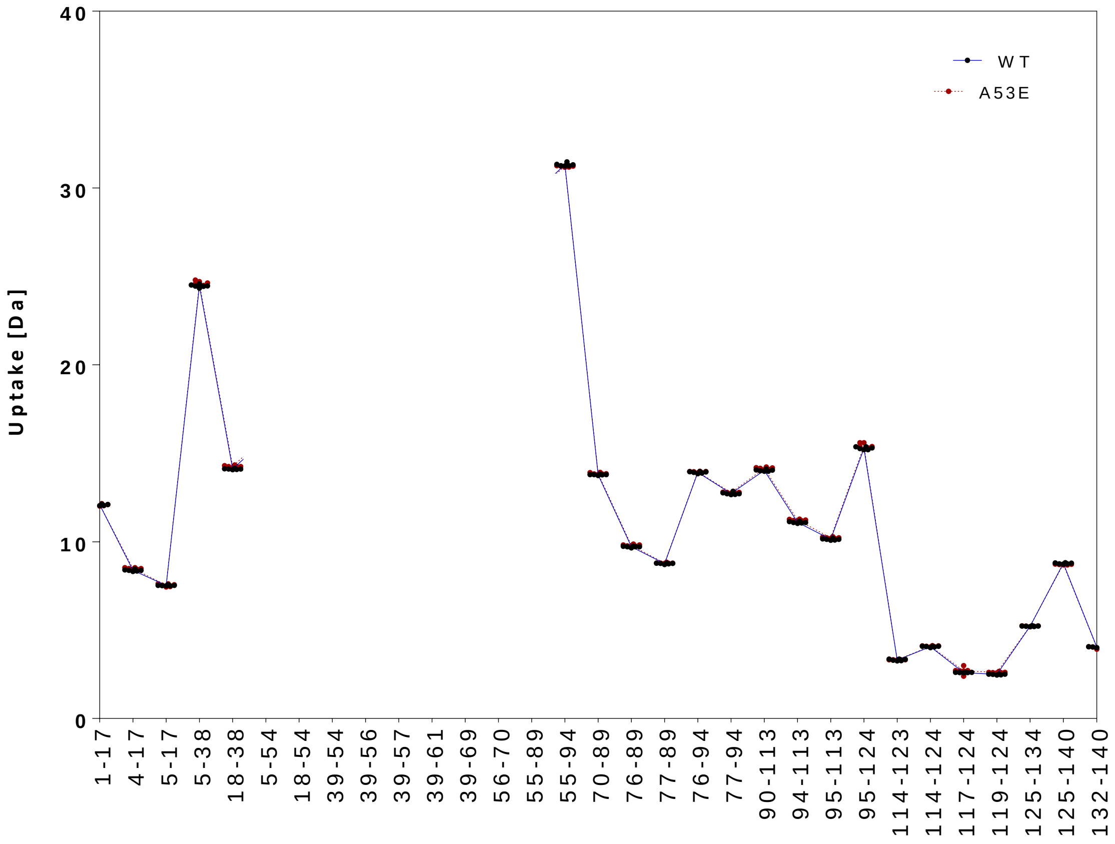

Supplementary Figure 26. Deuterium uptake plots, measured in Dalton (Da), for A. WT aSyn (blue) and A53T aSyn (red), and WT aSyn (blue) and A53E aSyn (red), showing the individual replicates for each measurement. Data correspond to manuscript main figures 6a and 6b.

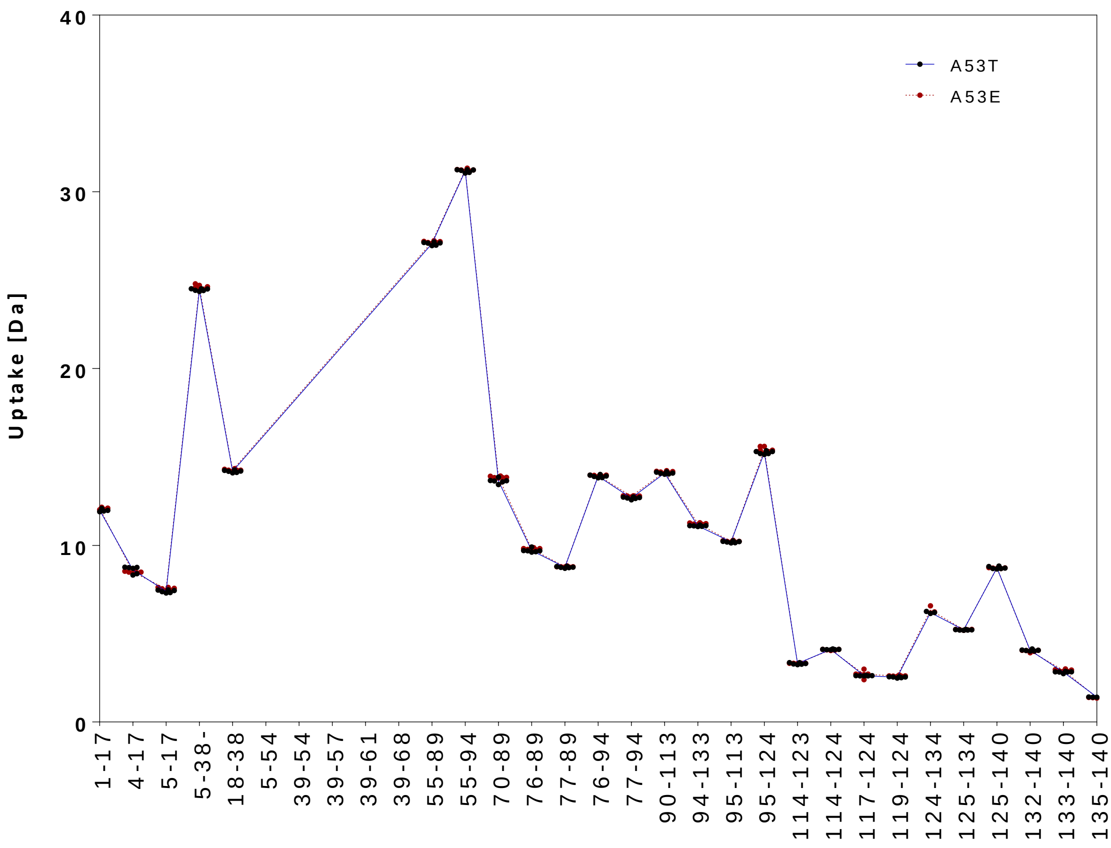

Supplementary Figure 27. Deuterium uptake plots, measured in Dalton (Da), for A53T aSyn (blue) and A53E aSyn (red), showing the individual replicates for each measurement. Data correspond to manuscript main figure 6c.

Supplementary Methods

Atomic Force Microscopy (AFM)

The contents of wells from the ThT-based assays were centrifuged for 20 minutes at 21 k x g. The supernatant was removed to keep 10 µL with remaining fibrils. The fibrils were resuspended and incubated on a freshly cleaved mica surface, which had been coated in 0.1% poly-l-lysine, for 20 min. The mica was washed three times in 18.2 Ω dH_2O to remove lose protein. Images were acquired in dH_2O using tapping mode on a BioScope Resolve (Bruker, AXS GmBH) using ScanAsyst-Fluid+ probes. 512 lines were acquired at a scan rate of 1.5-2 Hz per image with a field of view of 2-5 µm and for at least four fields of view. Images were adjusted for contrast and exported from NanoScope Analysis 8.2 software (Bruker).

Nano electrospray ionisation ion mobility mass spectrometry (Nano ESI-IM-MS)

A final concentration of 20 µM aSyn (WT or mutant) was obtained in 20 mM ammonium acetate (Sigma Aldrich, St. Louis, MO, USA) pH 7 and measured as a control. $CaCl_2$ (Merck, Darmstadt, Germany) was dissolved in deionised H_2O and added to the sample with a final concentration ranging between 200 µM and 400 µM. The samples were incubated for 10 minutes at room temperature before measuring. Nano-ESI (ion mobility-) mass spectrometry (nano-ESI-IM-MS) measurements were performed on a Synapt G2 HDMS (Waters, Manchester, U.K.) and analysed using Masslynx version 4.1 (Waters, Manchester, U.K.). For infusion into the mass spectrometer, home-made gold-coated borosilicate capillaries were used. The main instrumental settings were: capillary voltage 1.4-1.8 kV; sampling cone 25 V; extraction cone 1 V; trap CE 4 V; transfer CE 0 V; trap bias 40 V. Gas pressures used throughout the instrument were: source 1.5-2.7 mbar; trap cell 2.3 x 10^{-2} mbar; IM cell 3.0 mbar; transfer cell 2.5 x 10^{-2} mbar. p values calculated using an unpaired T-test without assuming equal variance.

Supplementary Table 13. Sequences of primers used in this study

Primers	5' to 3'
D115A Forwards	GAAGGAATTCTGGAAGCGATGCCTGTGGATCCTG
D115A Reverse	CAGGATCCACAGGCATCGCTTCCAGAATTCCTTC
D119A Forwards	GATATGCCTGTGGCGCCTGACAATGAGGC
D119A Reverse	GCCTCATTGTCAGGCGCCACAGGCATATC
D121A Forwards	CCTGTGGATCCTGCCAATGAGGCTTATG
D121A Reverse	CATAAGCCTCATTGGCAGGATCCACAGG
A30P Forwards	GTGGCAGAAGCACCCGGAAAGACAAAAG
A30P Reverse	CTTTTGTCTTTCCGGGTGCTTCTGCCAC
E46K Forwards	CAGCAGGAAAGACAAAAAAGGGTGTTCTCTATGTAG
E46K Reverse	CTACATAGAGAACACCCTTTTTTGTCTTTCCTGCTG
A53T Forwards	GGTGTGGCAACAACCGCTGAGAAGACC
A53T Reverse	GGTCTTCTCAGCGGTTGTTGCCACACC
A53E Forwards	GTGCATGGTGTGGAAACAGTGGCTGAG
A53E Reverse	CTCAGCCACTGTTTCCACACCATGCAC
H50Q Forwards	GAGGGAGTGGTGCAAGGTGTGGCAACAGTG
H50Q Reverse	CACTGTTGCCACACCTTGCACCACTCCCTC
G51D Forwards	GGAGTGGTGCATGATGTGGCAACAGTG
G51D Reverse	CACTGTTGCCACATCATGCACCACTCC

Supplementary References

1. Allison, J. R., Rivers, R. C., Christodoulou, J. C., Vendruscolo, M. & Dobson, C. M. A Relationship between the Transient Structure in the Monomeric State and the Aggregation Propensities of α-Synuclein and β-Synuclein. 53, 7183 (2014).

2. Wongkongkathep, P. et al. Native Top-Down Mass Spectrometry and Ion Mobility MS for Characterizing the Cobalt and Manganese Metal Binding of α-Synuclein Protein. J. Am. Soc. Mass Spectrom. 29, 1870–1880 (2018).

3. Han, J. Y., Choi, T. S. & Kim, H. I. Molecular Role of Ca2+ and Hard Divalent Metal Cations on Accelerated Fibrillation and Interfibrillar Aggregation of α-Synuclein. Sci. Rep. 8, 1895 (2018).
